# A new gene family (BAPs) of *Cotesia* bracovirus induces apoptosis of host hemocytes

**DOI:** 10.1080/21505594.2023.2171691

**Published:** 2023-02-07

**Authors:** Ze-Hua Wang, Xi-Qian Ye, Xiao-Tong Wu, Zhi-Zhi Wang, Jian-Hua Huang, Xue-Xin Chen

**Affiliations:** a Institute of Insect Science, Zhejiang University, Hangzhou, China; b Ministry of Agriculture Key Lab of Molecular Biology of Crop Pathogens and Insects, and Zhejiang Provincial Key Lab of Biology of Crop Pathogens and Insects, Zhejiang University, Hangzhou, China; c Regional Development and Governance Center, Hangzhou, China; d Guangdong Laboratory for Lingnan Modern Agriculture, Guangzhou, China; e State Key Lab of Rice Biology, Zhejiang University, Hangzhou, China

**Keywords:** Polydnavirus, *cotesia vestalis* bracovirus, host immunity, novel gene, apoptosis, *Plutella xylostella*

## Abstract

Polydnaviruses (PDVs), obligatory symbionts with parasitoid wasps, function as host immune suppressors and growth and development regulator. PDVs can induce host haemocyte apoptosis, but the underlying mechanism remains largely unknown. Here, we provided evidence that, during the early stages of parasitism, the activated *Cotesia vestalis* bracovirus (CvBV) reduced the overall number of host haemocytes by inducing apoptosis. We found that one haemocyte-highly expressed CvBV gene, *CvBV-26-4*, could induce haemocyte apoptosis. Further analyses showed that *CvBV-26-4* has four homologs from other *Cotesia* bracoviruses and BV from wasps in the genus *Glyptapanteles*, and all four of them possessed a similar structure containing 3 copies of a well-conserved motif (Gly-Tyr-Pro-Tyr, GYPY). Mass spectrometry analysis revealed that CvBV-26-4 was secreted into plasma by haemocytes and then degraded into peptides that induced the apoptosis of haemocytes. Moreover, ectopic expression of *CvBV-26-4* caused fly haemocyte apoptosis and increased the susceptibility of flies to bacteria. Based on this research, a new family of bracovirus genes, Bracovirus apoptosis-inducing proteins (*BAPs*), was proposed. Furthermore, it was discovered that the development of wasp larvae was affected when the function of CvBV BAP was obstructed in the parasitized hosts. The results of our study indicate that the *BAP* gene family from the bracoviruses group is crucial for immunosuppression during the early stages of parasitism.

## Introduction

The endoparasitoid wasps have adapted various methods to adjust their host’s physiology and development to guarantee the survival of wasp offspring [1,2]. Haemocytes play important roles in the immune responses of insects against parasites [3,4]. Therefore, parasitoid wasps induce a series of changes in host haemocytes to avoid haemocyte-mediated immune responses by polydnavirus (PDV) and venom, which are injected into host larvae along with parasitoid eggs during oviposition. For example, *Cotesia vestalis* parasitization inhibits recognition and encapsulation of the host haemocytes [5]; *Diadegma semiclausum* parasitization affects haematopoietic regulation and haemocyte-mediated immune responses in host larvae [6]; *Cotesia kariyai* parasitization reduces the total haemocyte count (THC) of the hosts [7]; The parasitization of *Pimpla turionellae* and *C. vestalis* alter the haemocyte population of their hosts and even the functions of the host haemocytes [8,9]. Among the changes of haemocyte-mediated immune responses post parasitization, apoptosis of haemocytes plays an important role, which was induced by PDVs [10–12].

PDV virion particles contain multiple segments of double-stranded, superhelical DNAs and form a mutually beneficial relationship with parasitoid wasps [13]. PDVs were classiﬁed into two genera: bracoviruses (BVs) and ichnoviruses (IVs) [14]. PDV virions infect nearly all host tissues but the haemocytes are the most heavily infected cells [15–17]. The DNAs of PDV are integrated chromosomally into host haemocytes [18–20]. Their viral genes are then transcribed in the affected host cells [15,21], resulting in the production of virulence proteins [22,23] or microRNAs [24].

The genome of eight BVs [25–32] and five IVs [29,33–36] have been fully sequenced and several PDV gene families were identified [37]. Gene families such as protein tyrosine phosphatases (PTPs), ankyrin-motif proteins, cys-motif proteins, and BEN-domain proteins were conserved among BVs [38]. PTPs act by dephosphorylating target proteins [2]. Ankyrins, which resemble *Drosophila melanogaster* NF-κb transcription factor inhibitors, block host immune responses, induce or inhibit the apoptosis of insect cells, and disrupt ecdysone biosynthesis [39–41]. BEN-domain proteins play a crucial role in suppressing haemocyte nodule formation [42]. In addition to the conserved gene families, BV genomes contain many genes that code for hypothetical proteins that lack the conserved domains to predict the functions [38]. Thus, the functions of many PDV genes remain unknown.

This paper aims to reveal the functional PDV gene(s) responsible for host haemocyte apoptosis. Herein, it is demonstrated that *C. vestalis* reduced the host THC by apoptosis induced by *C. vestalis* BV (CvBV). The global transcriptome statistics of CvBV genes in host haemocytes showed that CvBV-26-4 is secreted by haemocytes and degraded into functional peptides that induced haemocyte apoptosis. Further analysis displayed that *CvBV-26-4* and its analogous proteins from other BVs all contain 3 well-conserved motifs, Gly-Tyr-Pro-Tyr and function in inducing apoptosis. Hence, a new PDV gene family, *B**racovirus* *A**poptosis-inducing* *P**rotein* (*BAP*), was suggested for these proteins. Ectopic expression of CvBV *BAP* induced apoptosis of fly haemocytes and increased susceptibility of male flies to *Staphylococcus aureus*. Knockdown of *BAP* had a deleterious impact on the development of *C. vestalis* progeny. Thus, we inferred that the BV BAP family is important for host immunosuppression during parasitization.

## Materials and methods

### Insects

*P. xylostella* and *C. vestalis* were maintained in our lab as described in a previous study [43]. *Drosophila melanogaster* stocks were raised on standard cornmeal/yeast/agar medium at 18°C. In this study, *W*^*1118*^ was used as the wild-type stock and Bloomington stock *Hml-GAL4* (BS#8700) was also used.

### Haemocyte viability and counts

About 30 parasitized or non-parasitized *P. xylostella* haemocytes were collected. Cell viability and number counts were determined by trypan blue staining using Cell Counter (Countstar, Shanghai, China). Hemocyte counting was done 4 h post parasitization (pp). Cell viability was determined at 6 h, 2 h, and 4 h pp. 16 repeats (collections of 30 *P. xylostella* haemocytes) of each group were determined.

### Virus and venom collection

From the female wasps, CvBV virions were collected and incapacitated using UV light as described in a previous study [44]. *C. vestalis* venom gland was dissected and the stored venom protein was extracted. We defined the quantity of CvBV or venom from one female as one female equivalent (FE).

### Injection

The third instar larvae of *P. xylostella* (2 h post-ecdysis) and male flies (7 days post-eclosion) were used for injection of different fluids as previously described [44].

### Apoptosis detection

Haemocytes collected from 30 *P. xylostella* after different treatments or *Drosophila* larvae were suspended in 00 μL phosphate buffered saline (PBS, pH 7.4) and plated into the Lab-Tek II Chambered Cover glass (Thermo Fisher, MA, USA) for 30 min at room temperature. Apoptotic haemocytes were visualized using a terminal deoxynucleotidyl transferase dUTP nick end labelling (TUNEL) assay kit (Vazyme, Nanjing, China) according to guidelines. And 4, 6-diamidino-2-phenylindole (DAPI) (Sigma-Aldrich) was used for nuclei counterstaining. The images were captured using a confocal microscope, Zeiss LSM 800. To quantify the percentage of apoptotic haemocytes from *P. xylostella* and *D. melanogaster*, the labelled nuclei of > 3 images were counted using the Image J software.

### Caspase activity assay

After 4 h post treatment, the haemocytes were collected from *P. xylostella*. And the Caspase-Glo® 3/7 assay Kit (Promega, USA) was used to perform the Caspase activity assay. Each group contains 15 biological replicates.

### RNA extraction

To analyse gene expression at different times pp, we extracted the total RNA of *P. xylostella* at 0.5 h, 1 h, 2 h, 4 h, 8 h, 6 h, 4 h, 8 h, 2 h, 6 h, and 20 h pp. *P. xylostella* were dissected in PBS (pH 7.4) at 4 h pp and their tissues were collected to analyse gene expression in different tissues: silk gland, epidermis, midgut, fat body, central nervous system (CNS), haemocytes, testis, and malpighian tubule (MT). Total RNA was isolated using the Trizol method (Invitrogen, Carlsbad, CA, USA).

### mRNA sequencing and data analysis

The haemocytes of *P. xylostella* after being parasitized for 6 h, 2 h, and 4 h were collected and subject to transcriptome sequencing. In detail, each group contained 3 biological replicates in the form of 200 parasitized *P. xylostella*. For each library preparation, a total of 1 μg RNA was used as input material. And the VAHTS mRNA-seq v2 Library Prep Kit (Vazyme, Nanjing, China) was used for library generation. Clustering of the index-coded samples was performed on a cBot Cluster Generation System (Illumina, USA) according to guidelines. Then, the sequencing was performed on an Illumina HiSeq X Ten platform using the 150 bp paired-end module.

Paired-end clean reads were mapped to the CvBV genome [26] with TopHat (v2.1.1) [45] using the CvBV genome index created by Bowtie (v2.1.0) [46]. Raw sequencing data are available in the SRA database of NCBI (SRR10863199-SRR10863201). Cuffdiff (v2.2.1) [47] was used to calculate fragments per kilobase of exon per million fragments mapped (FPKMs) for coding genes in each group.

### Quantitative PCR

Quantitative PCR (qPCR) was used to validate the transcriptome data and study the expression proﬁles of selected genes in varying developmental stages and tissues. qPCR was performed as previously described [48]. *β-actin* gene (GenBank accession No. AB282645) and *β-tubulin* gene (GenBank accession No. EU127912) of *P. xylostella* were used as internal controls. The relative expression levels were calculated using the 2^−ΔΔCt^ method [48].

### Sequence analysis

A homolog search was carried out against other species using BLASTP (http://www.ncbi.nlm.nih.gov/), with CvBV-26-4 as the seed sequence. The signal peptide of CvBV-26-4 was predicted by SIGNALP 6.0 (http://www.cbs.dtu.dk/services/SignalP/). MEGA 7.0 software was used to perform the alignment analyses. The figures of the sequence analysis were created using Jalview [49].

### RNA silencing

The T7 RiboMAXTM Express kit (Promega, USA) was used to synthesize double-stranded RNA (dsRNA) of the *CvBV-26-4* gene and *GFP* gene. Primers used for dsRNA synthesis are displayed in Table S7. Before parasitization, 500 ng of ds*CvBV-26-4* was injected into the middle 3^rd^ instar larvae of *P. xylostella*, and ds*GFP* was used as the negative control. The efficacy of the RNA interference was determined by qPCR at 4 h pp.

### Recombinant baculovirus concentration

The vector, pFASTBAC-HTb (Invitrogen, CA, USA), was used for baculovirus expression in *P. xylostella* haemocytes. We modified pFASTBAC-HTb by inserting the open reading frames (ORFs) of *GFP*, *CvBV-2-1, CvBV-3-5*, and *CvBV-26-4*. Whole cDNA sequences of *CCQ71098.1* from CcBV and *CCQ19291.1* from *C. sesamiae Kitale* bracovirus (CsKBV) were synthesized by a simplified gene synthesis method. *CCQ71098.1* and *CCQ19291.1* were also inserted into the pFASTBAC-HTb. The production and concentration of recombinant baculovirus were performed as previously described [21,50].

### Western blot

A polyclonal antiserum against CvBV-26-4 was generated by the Shanghai Sangon Biotechnology Company using a peptide antigen for antiserum development. Epitopes are predicted by the GenScript Optimum Antigen design tool. The peptide sequence synthesized for the CvBV-26-4 antiserum development was YPYPSSDGSSGYFS.

Haemocytes and plasma of *P. xylostella* were separated by centrifugation. After adding SDS PAGE Loading Buffer, the samples were boiled for 0 min to denature the proteins. The western blot was performed as previously described [21]. In this study, polyclonal antiserum against CvBV BAP (1:500) and actin (1:3000), and secondary antibody against rabbit IgG (1:3000) were used. The ECL western blotting substrate (Promega, USA) was used to visualize the bands.

### Mass spectrometry of plasma peptides

To confirm whether CvBV-26-4 protein was secreted outside haemocytes and degraded into peptides, we collected peptides from the plasma of *P. xylostella*.~0 μl of haemolymph from 4 h pp *P. xylostella* larvae was added to 00 μl anticoagulant buffer [44]. The diluted haemolymph was centrifuged at 000 g at 4 ℃ for 5 min to obtain the plasma in the supernatant. The peptides from the plasma were isolated with a 3 kDa Millipore ultrafiltration device. The peptide mixtures were subjected to mass spectrometry analysis using a Thermo Scientific Easy nanoLC 1000 (Thermo Fisher Scientific, MA, USA). Peptide-spectrum matches (PSMs) were set to 10.

### Ectopic expression of CvBV-26-4 in drosophila melanogaster

TheORF sequence of *CvBV-26-4* was cloned into the pUAST-attb vector [51] to obtain the CvBV BAP transgenic lines. Integrase-mediated insertion by phiC31 into the attP2 landing-site locus on the 3^rd^ chromosome was used to obtain the transgenic *Drosophila* line carrying the *UAS-BAP* gene.

The survival assay was conducted as described [52]. Briefly, each male fly at 7 days post-eclosion was injected with 40 nL of the *Staphylococcus aureus* resuspension using an Eppendorf Femtojet (Eppendorf, Germany). The death was recorded every 2 h; flies that died within the first 6 h were removed. Flies were kept at 25°C and were transferred to new food every day.

### CvBV-26-4 peptide rescue experiments

Each middle 3^rd^ *P. xylostella* larva was injected with 0.1 μg of CvBV-26-4 antiserum and 0.1 μg of CvBV-26-4 peptide, and then parasitized by *C. vestalis*. Haemocytes collected from 30 *P. xylostella* at 4 h pp were used for apoptosis detection, as mentioned in the apoptosis detection section.

### Determination of C. vestalis development

To detect the effects of CvBV-26-4 on *C. vestalis* offspring, 0.1 μg each of CvBV BAP antiserum and rabbit IgG (negative control) were injected into the middle 3^rd^ instar *P. xylostella*, which were then parasitized by *C. vestalis*. The body length of wasp pupae was measured one day after pupation. The rate of wasp pupa formation was recorded. The eclosion of *C. vestalis* was also noted following wasp larva pupation.

### Statistical analysis

All statistical analyses were performed using SPSS 20.0 software. Data were presented as means ± SD and were examined by the One-way ANOVA and Tukey’s test, with a *p*-value of 0.05 as the significance threshold. Different survival curves were compared using log rank tests. Chi-squared tests were used to assess the rate of wasp pupa formation and eclosion.

## Results

### Parasitization reduced the total count of host haemocytes through apoptosis

**Figure 1.**
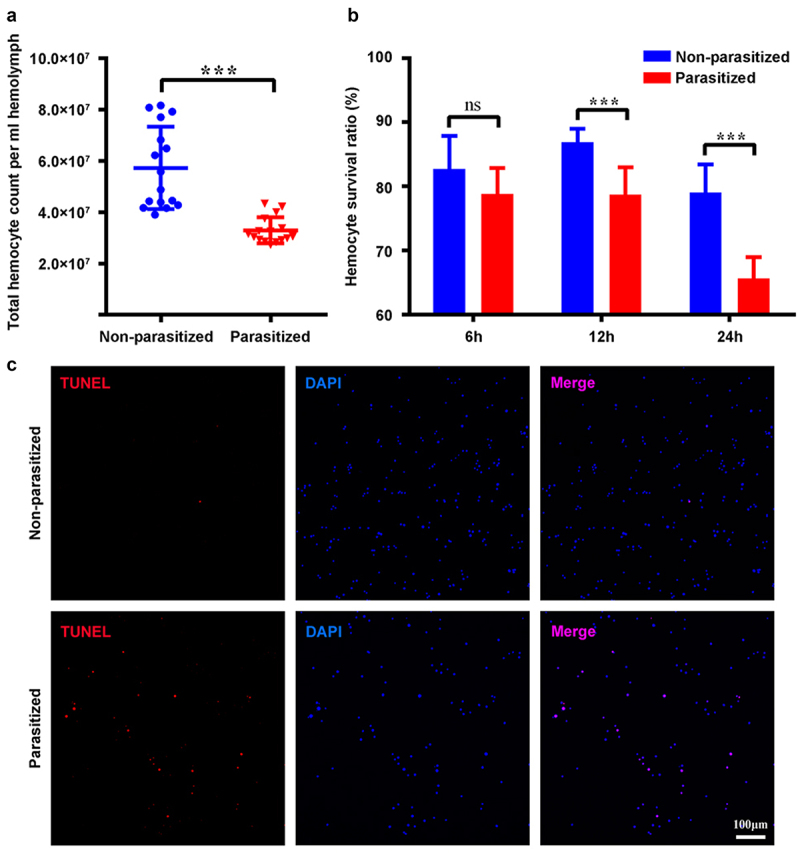
Parasitization reduced the total count of *P. xylostella* haemocytes via apoptosis. (a) the total haemocyte count (THC) of *P. xylostella* at 4 h pp by *C. vestalis*. (b) the survival ratio of *P. xylostella* haemocytes at 6 h, 2 h, and 4 h pp. Error bars indicate ± SD. Differences among samples were tested with tukey-test (ns: no signiﬁcance; *** *p*<0.001). (c) TUNELlabeling to observe changes of *P. xylostella* apoptotic haemocytes at 4 h pp. Hemocytes were dissected and isolated, nuclei were stained with DAPI (blue), and apoptotic cells were labelled by TUNEL (red) (scale bar = 0 μm).

We analysed the total number of haemocytes from parasitized host larvae at 4 h pp by trypan blue staining using Cell Counter. The results showed that the overall number of haemocytes of parasitized host larvae was less than that of the control group (Figure 1(a)). As the number of haemocytes decreased within 4 h pp, the survival ratio of haemocytes should be affected earlier after parasitization. As expected, the survival ratio of haemocytes started to be affected at 2 h pp (Figure 1(b)). The survival ratio of haemocytes from parasitized host larvae at 4 h pp was below 70% (Figure 1(b)). Additionally, the detection of apoptosis at 4 h pp by the TUNEL assay revealed many positive haemocytes from parasitized host larvae, whereas most haemocytes from non-parasitized host larvae were negative (Figure 1(c) and S1a). Furthermore, we detected the caspase activity of haemocytes from *P. xylostella* at 4 h pp and the result showed that parasitization induced the caspase activity of host haemocytes (Figure S2a).

### The activated CvBV-induced apoptosis of host haemocytes

**Figure 2.**
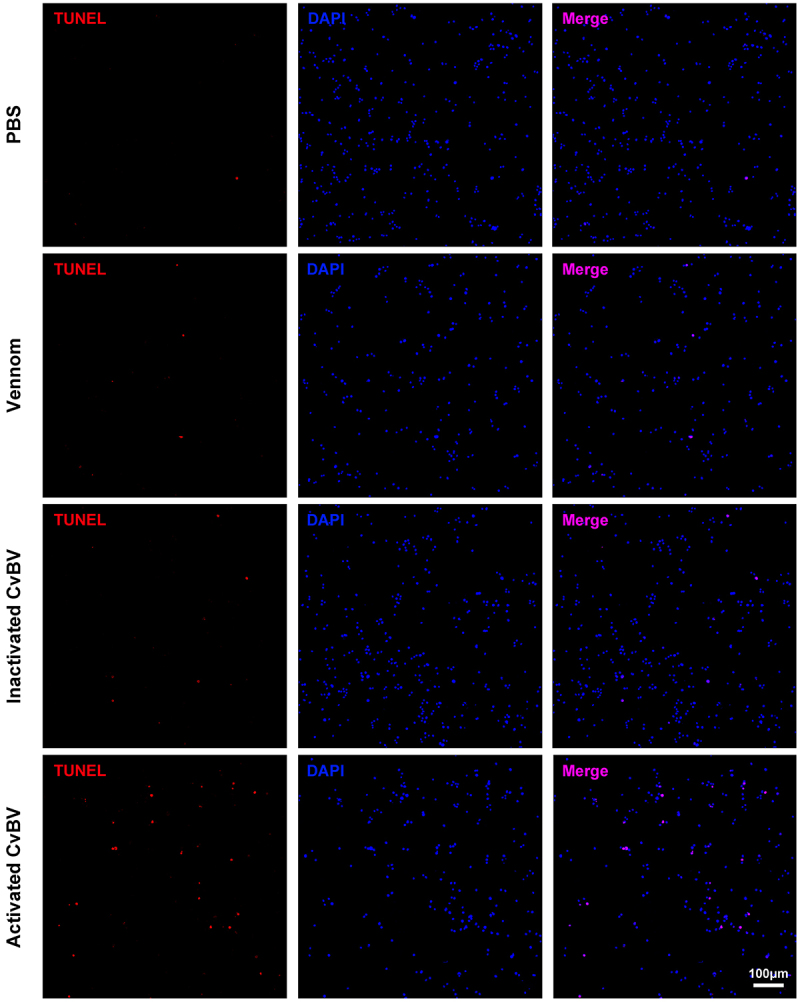
The activated CvBV induced apoptosis of host haemocytes. TUNEL apoptotic labelling to observe changes of apoptotic haemocytes of *P. xylostella* at 4 h post-injection of PBS, venom, inactivated and activated CvBV. The dosages of CvBV and venom were 0.05 FE. CvBV virions were put under UV-light for 2 hours to incapacitate the virus. Hemocytes were dissected and isolated, nuclei were stained with DAPI (blue), and apoptotic cells were labelled by TUNEL (red) (scale bar = 0 μm).

The injection of CvBV or venom into host larvae during wasp oviposition might induce apoptosis of host haemocytes. To evaluate the effects of CvBV and venom on the apoptosis of host haemocytes pp, we detected the apoptotic haemocytes of *P. xylostella* following CvBV and venom injection by a TUNEL assay. The results showed that 0.05 FE CvBV, a dosage close to that administered during real parasitism [44], induced apoptosis of haemocytes while 0.05 FE venom or PBS could not (Figure 2 and S1b). Further analyses indicated that apoptosis of host haemocytes was not induced by 0.05 FE inactivated CvBV (Figure 2 and S1b), which suggested that apoptosis was induced by virulent CvBV instead of its capsid proteins.

### Global transcriptome analysis of CvBV genes in P. xylostella haemocytes

RNA-seq analyses of host haemocytes were performed to elucidate genes or gene families of CvBV that induce apoptosis of host haemocytes. The target gene or genes should be abundantly expressed before 2 h pp, at which time the survival ratio of haemocytes is affected (Figure 1(b)). The Illumina HiSeq X Ten platform was used to sequence the mRNA of haemocytes from host larvae at 6 h, 2 h, and 4 h pp to obtain the gene expression profiles. A total of 537,693,406 clean reads were obtained from 9 cDNA libraries (Table S8). The lowest number of clean reads obtained from sample 6 h-3 nearly reached 55 million, and the highest number of reads obtained from sample 4 h-1 was more than 67 million. A total of 16,810,694 reads (3.13%) corresponding to the CvBV genome were obtained from 9 libraries (Table S1).

**Figure 3.**
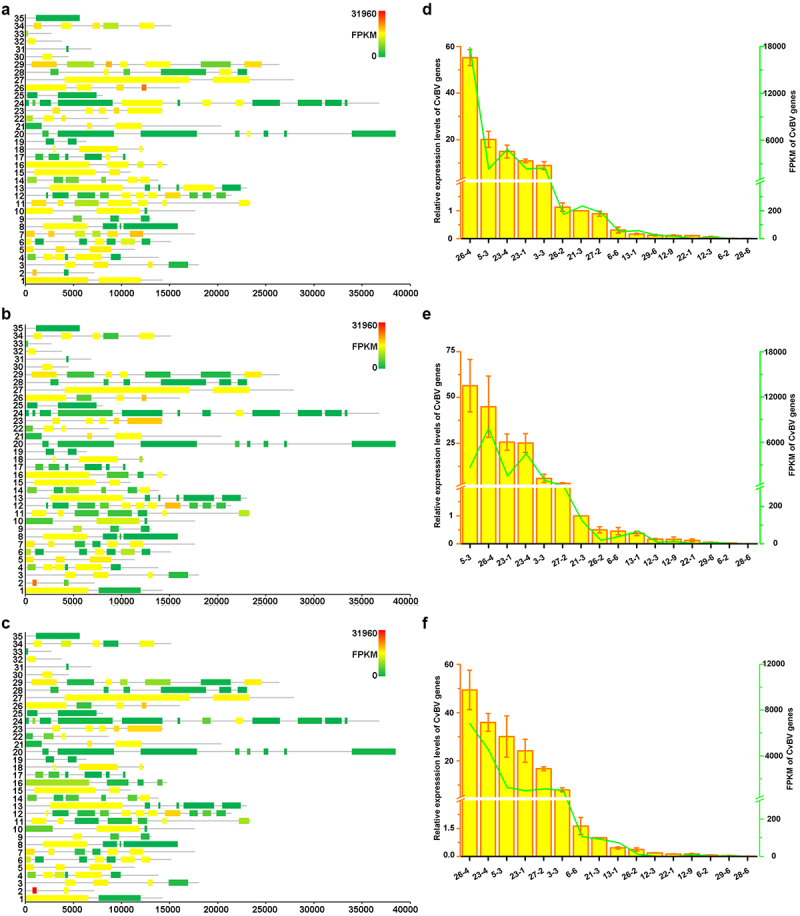
Expression levels of CvBV genes in *P. xylostella* haemocytes. CvBV gene expression levels in *P. xylostella* haemocytes at 6 h (a), 2 h (b), and 4 h (c) pp by *C. vestalis*. The transcripts were classiﬁed according to their location in the genome. 35 non-redundant circular CvBV genome was represented as linear molecules to visualize size and scale bar is in bps. For each circle, grey lines represent intergenic regions and different colours indicate the FPKMs of CvBV genes (see the list in S9, S10, and S11 Tables). Relative expression levels of CvBV genes in *P. xylostella* haemocytes were analysed using qPCR at 6 h (d), 2 h (e), and 4 h (f) pp. The right y-axis represents their FPKMs (green line) based on transcriptome data and the left ordinate represents relative expression levels (yellow columns). Error bars indicate ± SD.

The dynamic changes of CvBV gene expression profiles at different times pp were analysed. Among 157 predicted CvBV genes [26], 140 were detected in haemocytes at 6 h, 2 h, and 4 h pp. Their FPKMs are shown in Tables S9, S10, and S11. Global transcriptome statistics of CvBV gene transcripts showed that the relative expression levels of various CvBV genes differed at different times pp (Figure 3(a–c)). Based on the differences between gene expression levels in haemocytes, CvBV genes were classiﬁed into 4 categories, high (FPKM≥1000), medium (1000>FPKM≥100), low (100>FPKM≥10), and marginal (10>FPKM>0). To validate the results analysed from transcriptomes, 16 CvBV genes were randomly selected across the observed range of expression for qPCR analysis of the cDNA samples from haemocytes used for sequencing. These qPCR analyses conﬁrmed the trends observed from transcriptome analyses (Figure 3(d–F)). The specific number of CvBV genes expressed in host haemocytes at 6 h, 2 h, and 4 h pp was 129, 124, and 133, respectively (Figure S3a). There were 14 CvBV genes expressed in haemocytes at a high level within the observed 4 h pp (Figure S3b).

### CvBV-26-4 induced apoptosis of P. xylostella haemocytes

**Figure 4.**
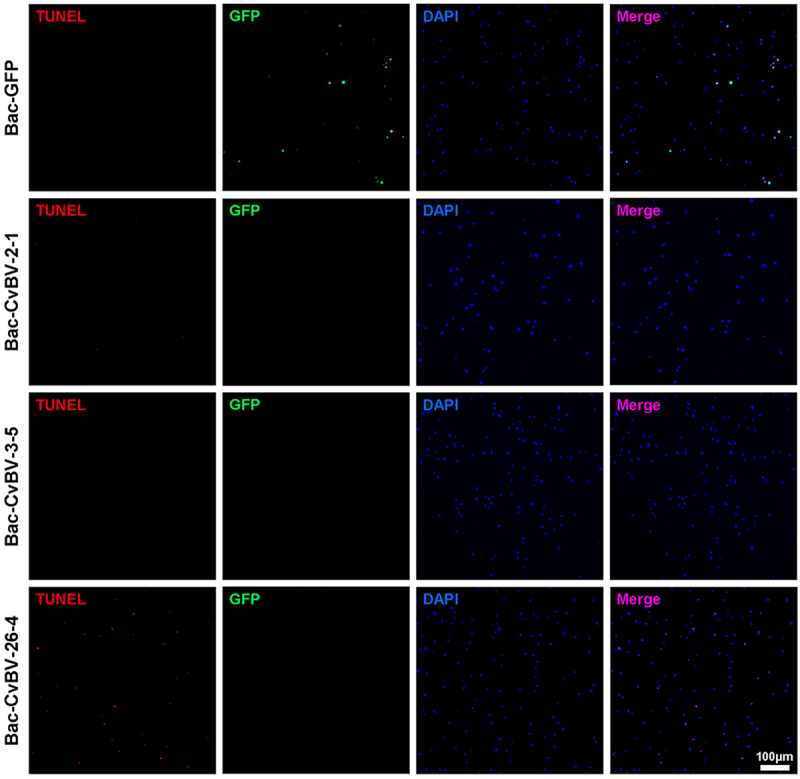
Overexpression of *CvBV-26-4* induced apoptosis of *P. xylostella* haemocytes. The TUNEL apoptotic labelling to observe changes of haemocytes infected by modified baculoviruses (Bac-GFP, Bac-CvBV-2-1, Bac-CvBV-3-,5 and Bac-CvBV-26-4). Hemocytes were dissected and isolated, nuclei were stained with DAPI (blue), and apoptotic cells were labelled by TUNEL (red) (scale bar = 0 μm).

Among the top 10 CvBV genes highly expressed in host haemocytes at 6 h pp, *CvBV-2-1*, *CvBV-3-5*, and *CvBV-26-4* were the three most highly expressed within 4 h pp (Figure S4), indicating that these three genes might be involved in the apoptosis of host haemocytes. To figure out which gene was involved in inducing apoptosis of host haemocytes, *CvBV-2-1* (GenBank accession No. AEE09453.1), *CvBV-3-5* (GenBank accession No. GU299780.1), and *CvBV-26-4* (GenBank accession No. AEE09579.1) were firstly cloned from parasitized host larvae, and 10^4^ pfu baculoviruses inserted with *GFP*, *CvBV-2-1*, *CvBV-3-5*, and *CvBV-26-4* were then injected into middle 3^rd^ instar *P. xylostella*, respectively. Host haemocytes at 4 h post-injection (pi) were used for apoptosis detection. The results showed that only *CvBV-26-4* was involved in the apoptosis of host haemocytes (Figure 4 and S1c).

**Figure 5.**
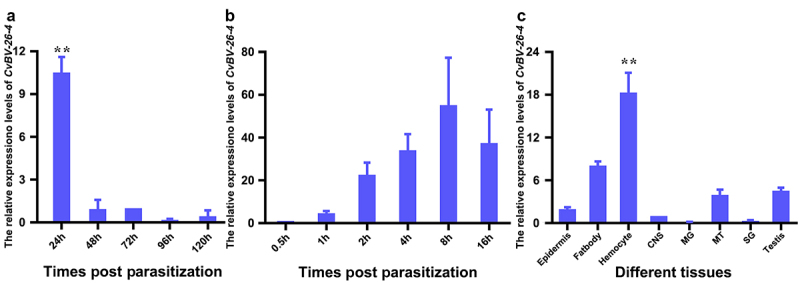
Expression profile of *CvBV-26-4* in *P. xylostella*. (a) *CvBV-26-4* expression profile in *P. xylostella* at 4 h, 8 h, 2 h, 6 h, and 20 h pp. (b) *CvBV-26-4* expression profile in *P. xylostella* at 0.5 h, 1 h, 2 h, 4 h, 8 h, and 6 h pp. (c) the expression profile of *CvBV-26-4* was measured by qPCR in 8 tissues of *P. xylostella* at 4 h pp including the epidermis, silk gland, fat body, midgut, haemocytes, central nervous system (CNS), malpighian tubules (MT), and testis. Error bars indicate ± SD (*n* = 3). Samples were compared with the tukey-test (**: indicates samples with a significant difference *p*<0.01).

Furthermore, the expression levels of *CvBV-26-4* in parasitized host larvae at different times were determined with the qPCR analysis. Our analyses showed that *CvBV-26-4* was expressed abundantly at 4 h pp and rapidly decreased by 8 h pp (Figure 5(a)). However, six more sample time points within 4 h pp were refined to determine the transcript profiles of *CvBV-26-4* at the earlier stage. *CvBV-26-4* expression level increased rapidly at 4 h pp (Figure 5(b)). qPCR analysis showed that the transcript level of *CvBV-26-4* was much higher in the haemocytes at 4 h pp than that in other tissues (Figure 5(c)), suggesting that *CvBV-26-4* plays an important role in the apoptosis of host haemocytes during the early stages of parasitism.

**Figure 6.**
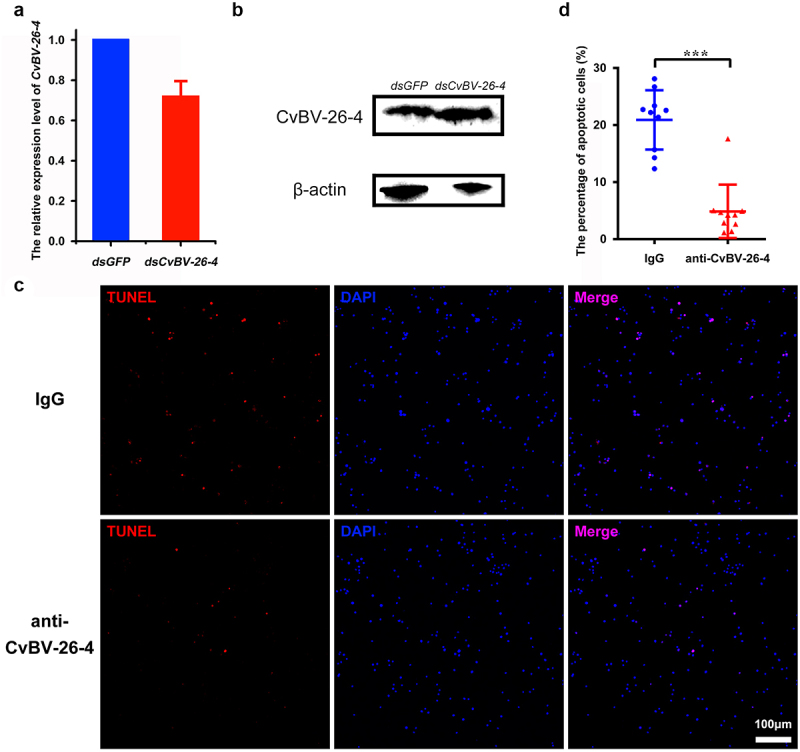
CvBV-26-4 is involved in the apoptosis of *P. xylostella* haemocytes. (a) the expression levels of *CvBV-26-4* were measured by qPCR 4 h after the interference of *CvBV-26-4* was introduced. Error bars indicate ± SD (*n* = 3). (b) Western blot of CvBV-26-4 in host haemolymph 4 h after the interference of *CvBV-26-4*. (c) TUNEL labelling of apoptotic haemocytes from *P. xylostella* at 4 h pi with rabbit IgG or CvBV-26-4 antiserum. Hemocytes were dissected and isolated, nuclei were stained with DAPI (blue), and apoptotic cells were labelled by TUNEL (red) (scale bar = 0 μm). (d) the percentage of apoptotic haemocytes from *P. xylostella* at 4 h post-injection of normal rabbit IgG or the CvBV-26-4 antiserum (*n* = 10). Differences among samples were tested with tukey-test (***: statistically significant *p*<0.001).

To further test whether the apoptosis was caused by *CvBV-26-4*, gene silencing was conducted via dsRNA injection, while it was discovered that neither the transcript level of *CvBV-26-4* nor its protein content was affected (Figure 6(a,b)and S5). Then, prior to parasitization, we interfered with CvBV-26-4 via injecting the CvBV-26-4 antiserum, an injection of rabbit IgG that served as a negative control. We detected fewer TUNEL positive haemocytes after injection of the CvBV-26-4 antiserum compared with negative control (Figure 6(c,d)), which suggests that CvBV-26-4 indeed caused the apoptosis of host haemocytes.

### CvBV-26-4 homologs induced apoptosis of P. xylostella haemocytes

**Figure 7.**
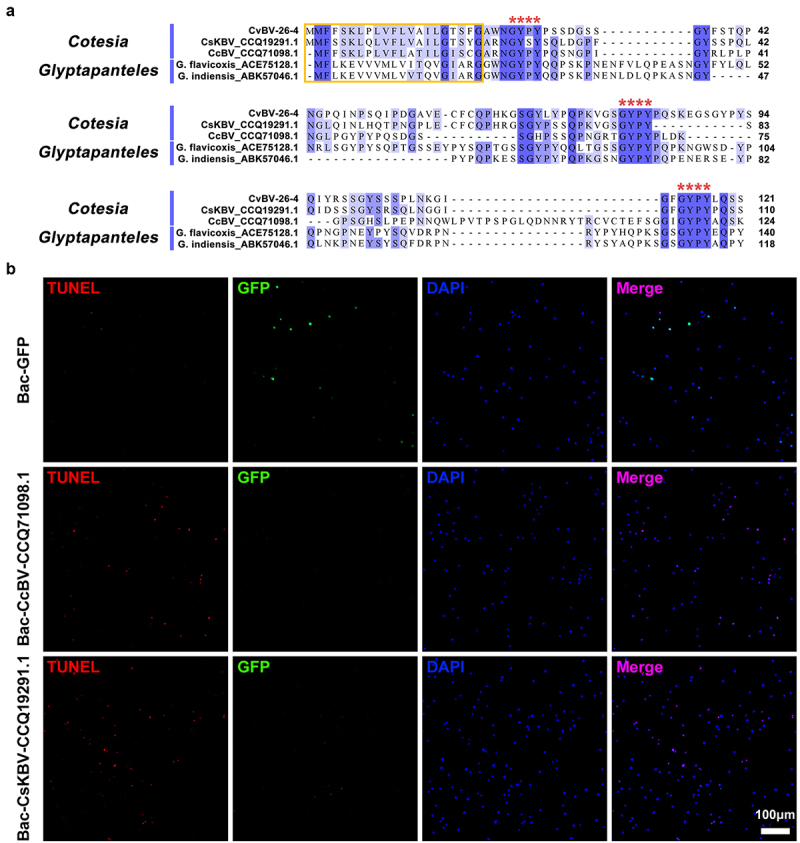
CvBV-26-4 homologs induced apoptosis of *P. xylostella* haemocytes. (a) amino acid sequence alignments of CvBV-26-4 and its homologs in *Cotesia* and *glyptapanteles*. The red box indicates the signal peptides. GYPY motifs conserved in CvBV-26-4 and its homologs are marked by red **** above the sequences. The numbers on the right are the position of the final amino acid. (b) the TUNEL apoptotic labelling of haemocytes at 4 h post-infection by modified baculoviruses (Bac-GFP, Bac-CcBV_CCQ71098.1, and Bac-CsKBV_CCQ19291.1). Hemocytes were dissected and isolated, nuclei were stained with DAPI (blue), and apoptotic cells were labelled by TUNEL (red) (scale bar = 0 μm).

When using BLASTP to conduct the homolog search, four homologs of CvBV-26-4 were identified: two homologs from *Cotesia* BV (GenBank accession No. CCQ19291.1 and CCQ71098.1) and two homologs from the genus *Glyptapanteles* (GenBank accession No. ACE75128.1 and ABK57046.1), BV-carrying wasps. We aligned the amino acid sequences of these homologs with CvBV-26-4 to clarify whether there are common structural features. The alignment showed that the selected homologs possessed the same signal peptide and 3 well-conserved Gly-Tyr-Pro-Tyr (GYPY) motifs (Figure 7(a)). To assess whether the two homologous proteins from *Cotesia* BV have a similar function to CvBV-26-4, 10^4^ pfu baculoviruses modified with the insertion of *GFP*, *CvBV-26-4*, *CcBV_CCQ71098.1*, and *CsKBV_CCQ19291.1* were injected into middle 3^rd^ instar *P. xylostella*, respectively. The expression of CvBV-26-4 was confirmed by western blotting (Figure S6). Host haemocytes at 4 h pi were observed for apoptosis detection and the results showed that *CvBV-26-4*, *CcBV_CCQ71098.1*, and *CsKBV_CCQ19291.1* can induce apoptosis of haemocytes while GFP cannot (Figure 7(b) and S1c). With common structural features and a similar function, these proteins were therefore named Bracovirus Apoptosis-inducing Proteins (BAPs).

### CvBV BAP peptide induced apoptosis of P. xylostella haemocytes

**Figure 8.**
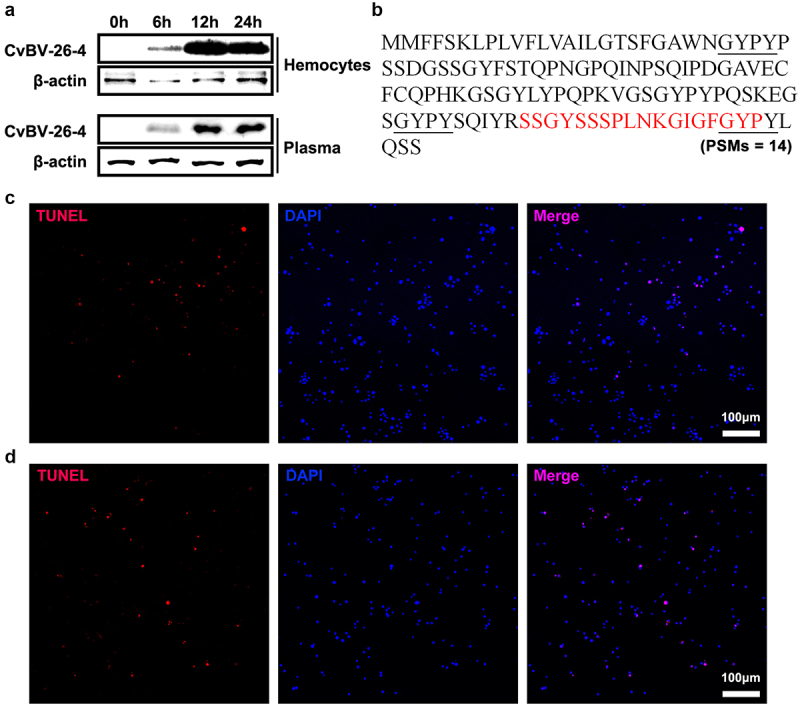
CvBV-26-4 peptide induced apoptosis of *P. xylostella* haemocytes. (a) amount of CvBV-26-4 protein in the haemocytes (top) and plasma (bottom) of *P. xylostella* at 6 h, 2 h, and 4 h pp. A band in the size range of 13 kDa that corresponds to the predicted CvBV-26-4 protein. Western blot are reported in supplementary information (figure S7). (b) the locations of CvBV 26–4 peptide (red) identified in the plasma of *P. xylostella* at 4 h pp by mass spectrometry. PSMs = 14. The conserved GYPY motifs were marked by underline. (c) the TUNEL apoptotic labelling of apoptotic haemocytes of *P. xylostella* at 4 h post-injection of the CvBV-26-4 peptide. (d) TUNEL labelling of apoptotic haemocytes in parasitized *P. xylostella* 4 h post-injection with CvBV-26-4 antibody and CvBV-26-4 peptide. Hemocytes were dissected and isolated, nuclei were stained with DAPI (blue), and apoptotic cells were labelled by TUNEL (red) (scale bar = 0 μm).

As CvBV BAP (CvBV-26-4) contains a signal peptide, it was hypothesized that CvBV BAP may be secreted and function outside of haemocytes. In the host haemocytes and plasma, CvBV BAP protein content was detected at 6 h, 2 h, and 4 h pp. The CvBV BAP proteins in the host haemocytes and plasma were detected at 6 h pp, accumulated rapidly by 2 h pp, and were maintained at a high level at 4 h pp (Figure 8(a) and S7). We wonder if the CvBV BAP proteins could be degraded into peptides in host plasma but not in host haemocytes. We collected peptides of host plasma at 4 h pp for mass spectrometry. One peptide of CvBV BAP (peptide-spectrum matches, PSMs = 14) was identified (Figure 8(b)). The CvBV BAP peptide contained 18 amino acids, SSGYSSSPLNKGIGFGYP, which is at the C terminus of the CvBV BAP protein. Apoptosis detection of host haemocytes at 4 h pi of BAP peptide showed that CvBV BAP peptide can induce apoptosis of haemocytes (Figure 8(c) and S1d). And the caspase activity of *P. xylostella* also increased post injection of the BAP peptide (Figure S2b). Injection of the CvBV BAP antiserum can rescue the apoptosis haemocytes from parasitized *P. xylostella* (Figure 6(d)), but injection of CvBV BAP antiserum can not rescue the apoptosis of haemocytes from parasitized *P. xylostella* when co-injected with the BAP short active peptide (Figure 8(d) and S1d).

### CvBV BAP induced apoptosis of D. melanogaster haemocytes

**Figure 9.**
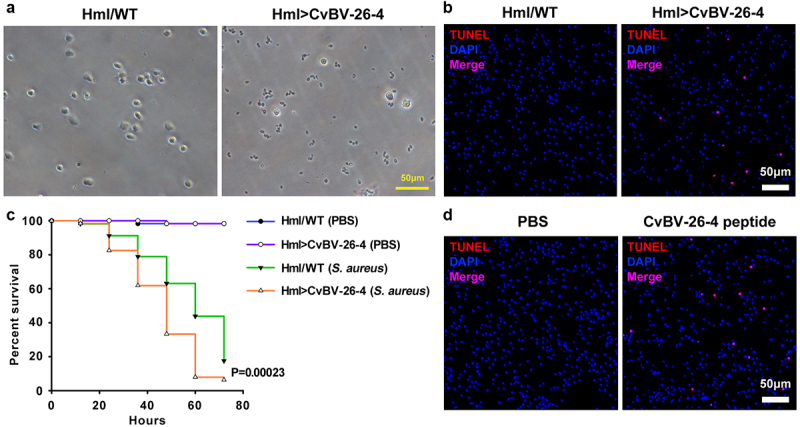
CvBV-26-4 induced apoptosis of *D. melanogaster* haemocytes. (a) morphologies of 3^rd^ instar fly haemocytes that ectopically expressed *CvBV-26-4* (scale bar = 0 μm). (b)apoptotic labelling of *Drosophila* larvae blood cells that ectopically expressed *CvBV-26-4*. Hemocytes were dissected and isolated, nuclei were stained with DAPI (blue), and apoptotic cells were labelled by TUNEL (red) (scale bar = 0 μm). (c) representative survival graphs of adult male *Drosophila* ectopically expressing *CvBV-26-4* after injection of *S. aureus* (OD600 0.4). *n* = 20–25 flies. Experiments were performed in triplicate. (d) apoptotic staining of haemocytes from 3^rd^ instar *Drosophila* larvae (*W*^*1118*^) at 4 h post-injection of 0.1 μg CvBV BAP peptide (injection of PBS was used as a negative control). Hemocytes were dissected out and nuclei were stained with DAPI (blue) and apoptotic cells were labelled by TUNEL (red) (scale bar = 0 μm).

To confirm the function of CvBV BAP proteins in non-adapted hosts, *D. melanogaster*, we used the GAL4/UAS binary expression system [53]. A *D. melanogaster* transgenic cell line carrying a UAS transgene encoding the CvBV BAP protein was produced. Then, we used a haemocyte specific expression driver, *Hml-GAL4*, to express *UAS-BAP* in *D. melanogaster* haemocytes [54]. Morphological observation showed that haemocytes from the 3^rd^ instar *Hml>BAP* larvae rarely keep normal forms (Figure 9(a)). The aberrant morphology phenotype was caused by apoptosis (Figure 9(b)). In addition, a survival assay was used to determine the effect of CvBV BAP on *D. melanogaster* haemocytes. The susceptibility to *S. aureus* of *Hml>BAP* flies was increased compared to controls (Figure 9(c) and S1e).

The TUNEL signal was detected in haemocytes from 3^rd^ instar *D. melanogaster* larvae 4 h post CvBV BAP (0.1 μg) injection showing that CvBV BAP can induce apoptosis of *D. melanogaster* haemocytes (Figure 9(d) and S1f).

### Effects of CvBV BAP on parasitization

**Figure 10.**
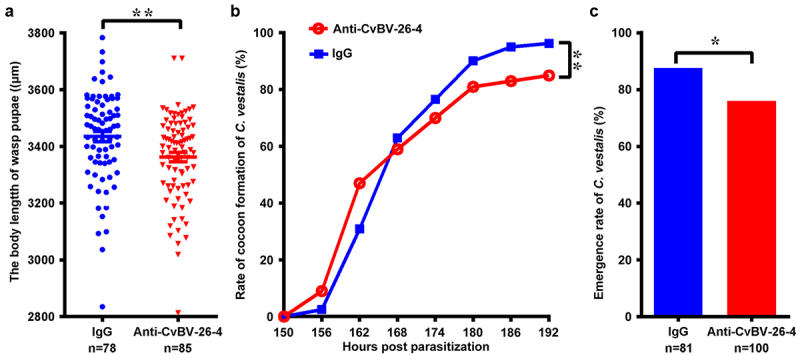
Interference of CvBV-26-4 on wasp development. 3^rd^ instar *P. xylostella* larvae were injected with CvBV-26-4 antibody or rabbit IgG before parasitization. (a) body lengths of 1-day wasp pupae (µm). Error bars indicate ± SD. Differences among samples were tested with tukey-test (**: significant difference between samples *p*<0.01). Rate of wasp pupa formation (b) and eclosion rate of *C. vestalis* (c) in 3^rd^ instar *P. xylostella* larvae injected with CvBV-26-4 antibody or rabbit IgG before parasitization. Differences among samples were tested with the chi-squared test. (*: significant difference between samples *p* <.

For detecting the effect of CvBV BAP on parasitization, the CvBV BAP antiserum was injected into the middle 3^rd^ instar larvae of *P. xylostella* which were then parasitized by *C. vestalis*. The body lengths of the wasp pupae, the wasp pupa formation, and the eclosion of *C. vestalis* were analysed to evaluate the wasp development. The results showed that the body lengths of wasp pupae were significantly decreased post-injection of BAP antiserum compared to the control group because of the blocked function of CvBV BAPs (Figure 10(a)). Injection of CvBV BAP antiserum reduced the percentage of wasp pupa formation (Figure 10(b)) as well as the eclosion rate of *C. vestalis* (Figure 10(c)).

## Discussion

The total number of haemocytes including granular cells and plasmatocytes decreased significantly in 4^th^ instar host larvae parasitized by *C. vestalis* [9]. This study found that parasitization affected the THC of *P. xylostella* much earlier in development. The total number of haemocytes from the late 3^rd^ host larvae at 4 h pp decreased and the survival ratio of haemocytes was affected at 2 h pp. It was similar to the reports indicating that the number of host haemocytes decreased during the early stages of parasitism by *C. melanoscela* [55], *Campoletis sonorensis* [56], *C. kariyai* [7], and *Pimpla turionellae* [8]. The THC of *P. xylostella* larvae was reduced because of apoptosis caused by parasitization, which was consistent with that found in *Pseudoplusia includes* [11] and *Spodoptera litura* [12]. Inducing apoptosis of host haemocytes is an important strategy for immunosuppression.

*C. vestalis* has three important wasp-associated factors which influence the physiology and development of host larvae, including CvBV, venom, and teratocytes [57]. Teratocytes are released into the host when the wasp egg hatches [58], while venom and PDV function earlier. Our results of different injections showed that the activated CvBV induced apoptosis of host haemocytes. In the CvBV genome, 157 ORFs were identified [26]. RNA-seq analyses of the host haemocyte transcriptomes showed that CvBV-26-4 might be involved in the induction of apoptosis of host haemocytes. It is not easy to silence *CvBV-26-4* via injection of dsRNA, so we injected its antiserum to block functional CvBV-26-4. As expected, apoptotic haemocytes were reduced by nearly 75% after injection of the CvBV antiserum, which indicated that CvBV-26-4 was one of the key genes inducing apoptosis during the early stage of parasitism.

CvBV-26-4 homologs were found in two *Cotesia* BVs and two *Glyptapanteles* wasps. The homologs from CcBV and CsKBV also induced apoptosis of *P. xylostella* haemocytes. Therefore, we named these proteins as Bracovirus Apoptosis-inducing Proteins (BAPs). These *BAP* genes may belong to a new gene family of BVs. It is not surprising that *BAPs* were found only in *Glyptapanteles* and *Cotesia* wasps because *Glyptapanteles* is the sister genus to *Cotesia* (Figure S8) [59], which suggests that other variants may exist given the species diversity in these two genera. We proposed that *BAPs* might have originated between 54 Mya and 17 Mya based on the phylogeny of the microgsterid lineage (Figure S8).

The transcript level of CvBV *BAP* in the haemocytes of parasitized *P. xylostella* at 6 h pp was higher than that at 2 h pp, but BAP protein content in the parasitized host haemocytes was most abundant at 2 h pp when the survival ratio of haemocytes started to be affected, which suggests that BAP proteins were accumulated. An unexpected finding was that CvBV BAP degraded into a functional peptide in host plasma. PDV proteins secreted by haemocytes into plasma could affect a greater number of haemocytes and possibly other tissues. For example, CvBV BAP peptides may be a ligand for some receptors, which can induce apoptosis. Since CvBV BAP peptide was detected in the plasma (Figure 8(b)), but not in the haemocytes (mass spectrometry analysis, data not shown), and the precursor of BAP has a predicted signal peptide, we hypothesized that the precursor of BAP was secreted into plasma and degraded into the active forms. The antiserum made against a different part of the BAP protein may bind BAP secreted into the haemolymph to prevent the processing of BAP into the active forms, which would explain why the antiserum is able to inhibit the protein’s function (Figure 6(c,d)). In our study, the level of baculovirus infection is not high, but Bac-CvBV-26-4-induced apoptosis was observed in a large number of host haemocytes, which confirms our hypothesis. It is worth noting that a fraction of CvBV BAP may get into plasma due to cell lysis for more apoptotic haemocytes post parasitization. Further work is required to confirm that the degradation happens in the haemolymph and is necessary for function, to determine which proteases cleave CvBV BAP, and to identify the receptor of the CvBV BAP peptide.

CvBV BAP can also induce apoptosis of *Drosophila* haemocytes, which suggests that CvBV BAP could affect some conserved factors in the apoptosis pathway. The development of wasp larvae in the host was affected when the function of CvBV BAP was blocked. The host immunity response increased at the early stage of parasitism for the blocking of CvBV BAP, which results in shorter body lengths of wasp pupae, a lower percentage of wasp pupation, and a reduced rate of pupa to adult eclosion.

In summary, a new gene family from BVs was identified: the *BAP* family. Genes from the *BAP* family encoded proteins containing GYPY motifs, as well as proteins that induced apoptosis of *P. xylostella* haemocytes. CvBV BAP was secreted into host haemocoel and degraded into functional peptides to induce apoptosis. The *BAP* gene family is important for the successful parasitization of *Cotesia* wasps for its function in immunosuppression.

## Supplementary Material

Supplemental Material

## Data Availability

The raw data for mRNA sequencing of *P. xylostella* haemocytes are deposited at SRA database of NCBI at https://www.ncbi.nlm.nih.gov, with accession number SRR10863199~SRR10863201.
