# Supplementary material for: A new gene family (BAPs) of Cotesia bracovirus induces apoptosis of host hemocytes
Source: Virulence. 2023 Feb 7;14(1):2171691. doi: 10.1080/21505594.2023.2171691 (PMC9908294; doi:10.1080/21505594.2023.2171691)
Supplement: Supplemental Material [file KVIR_A_2171691_SM5628.docx]

**Supplementary Materials**

**Table S1. The number of apoptotic hemocytes from *P. xylostella* at 24 h pp.**

| Replicate | Non-parasitized | |  | Parasitized | |  |
| --- | --- | --- | --- | --- | --- | --- |
|  | Apoptotic | Total |  | Apoptotic | Total | |
| 1 | 6 | 88 |  | 22 | 83 |  |
| 2 | 2 | 173 |  | 32 | 162 |  |
| 3 | 1 | 186 |  | 19 | 70 |  |
| 4 | 10 | 230 |  | 28 | 107 |  |
| 5 | 13 | 337 |  | 14 | 44 |  |

**Table S2. The number of apoptotic hemocytes from *P. xylostella* post different injections.**

| Replicate | PBS | |  | Venom | |  | Inactivated CvBV | |  | Activated CvBV | |
| --- | --- | --- | --- | --- | --- | --- | --- | --- | --- | --- | --- |
|  | Apoptotic | Total |  | Apoptotic | Total |  | Apoptotic | Total |  | Apoptotic | Total |
| 1 | 3 | 131 |  | 11 | 385 |  | 15 | 627 |  | 13 | 54 |
| 2 | 2 | 100 |  | 17 | 215 |  | 8 | 375 |  | 19 | 90 |
| 3 | 13 | 365 |  | 14 | 295 |  | 10 | 449 |  | 26 | 167 |
| 4 | 19 | 557 |  | 10 | 313 |  | 10 | 278 |  | 29 | 153 |
| 5 | 15 | 295 |  | 5 | 631 |  | 2 | 790 |  | 49 | 203 |

**Table S3. The number of apoptotic hemocytes from *P. xylostella* at 24 h post infected by modified baculoviruses.**

| Replicate | GFP | |  | CvBV-2-1 | |  | CvBV-3-5 | |  | CvBV-26-4 | |  | CcBV-CCQ71098.1 | |  | CsKBV-CCQ19291.1 | |
| --- | --- | --- | --- | --- | --- | --- | --- | --- | --- | --- | --- | --- | --- | --- | --- | --- | --- |
|  | Apoptotic | Total |  | Apoptotic | Total |  | Apoptotic | Total |  | Apoptotic | Total |  | Apoptotic | Total |  | Apoptotic | Total |
| 1 | 6 | 280 |  | 0 | 65 |  | 2 | 149 |  | 28 | 172 |  | 24 | 134 |  | 31 | 182 |
| 2 | 3 | 190 |  | 0 | 71 |  | 0 | 143 |  | 28 | 165 |  | 22 | 75 |  | 29 | 109 |
| 3 | 2 | 77 |  | 0 | 21 |  | 3 | 116 |  | 18 | 97 |  | 30 | 106 |  | 48 | 163 |
| 4 | 0 | 121 |  | 3 | 163 |  | 0 | 155 |  | 15 | 109 |  | 35 | 120 |  | 23 | 126 |
| 5 | 0 | 109 |  | 9 | 102 |  | 0 | 64 |  | 35 | 200 |  |  |  |  |  |  |

**Table S4. The number of apoptotic hemocytes from *P. xylostella* at 24 h post-injection of the CvBV-26-4 peptide and from parasitized *P. xylostella* at 24 h post-injection of the mixture of CvBV-26-4 antibody and CvBV-26-4 peptide.**

| Replicate | Non-parasitized **+** peptide | |  | Parasitized **+** antibody **+** peptide | |  |
| --- | --- | --- | --- | --- | --- | --- |
|  | Apoptotic | Total |  | Apoptotic | Total | |
| 1 | 47 | 186 |  | 60 | 409 |  |
| 2 | 33 | 180 |  | 46 | 240 |  |
| 3 | 38 | 190 |  | 59 | 286 |  |
| 4 | 52 | 199 |  | 28 | 183 |  |
| 5 | 46 | 271 |  | 44 | 178 |  |

**Table S5. The number of apoptotic hemocytes from *Drosophila* larvae that ectopically expressed *CvBV-26-4*.**

| Replicate | Hml/WT | |  | Hml>*CvBV26-4* | |
| --- | --- | --- | --- | --- | --- |
|  | Apoptotic | Total |  | Apoptotic | Total |
| 1 | 0 | 271 |  | 15 | 243 |
| 2 | 0 | 227 |  | 18 | 308 |
| 3 | 4 | 274 |  | 14 | 278 |
| 4 | 0 | 173 |  |  |  |
| 5 | 1 | 402 |  |  |  |

**Table S6. The number of apoptotic hemocytes from 3^rd^ instar *Drosophila* larvae (W1118) at 24 h post-injection of CvBV BAP peptide**

| Replicate | PBS | |  | Peptide | |
| --- | --- | --- | --- | --- | --- |
|  | Apoptotic | Total |  | Apoptotic | Total |
| 1 | 6 | 512 |  | 15 | 240 |
| 2 | 4 | 403 |  | 13 | 379 |
| 3 | 2 | 366 |  | 23 | 359 |
| 4 | 4 | 368 |  | 21 | 411 |
| 5 | 2 | 321 |  |  |  |

**Table S7 List of primers used in this study**

| **Primer name** | **Primer sequences (5’-3’)** | **Use** |
| --- | --- | --- |
| c12-3-rt-AP | ATCAGGAGATTTGTCTTCGGG | qPCR |
| c12-3-rt-P | GAAGAGTCACAACCAAGCAACAT | qPCR |
| c12-9-rt-AP | GATTTGTTATTAGGCATTCTGTTC | qPCR |
| c12-9-rt-P | TGTAGTAAGTGGGAAGTTCAAGATT | qPCR |
| c13-1-rt-AP | AATTTATGGCCCAGTCACGAG | qPCR |
| c13-1-rt-P | AATGTGCGGAGTAAACGAGAG | qPCR |
| c21-3-rt-AP | AACGTCACCAGAAGCTTGATCTA | qPCR |
| c21-3-rt-P | AACGATACAGTCCGTGGCAG | qPCR |
| c22-1-rt-AP | GTCTGTTAAATTCGGTGTGCTT | qPCR |
| c22-1-rt-P | GCCAATTATCCATCAAAGCG | qPCR |
| c23-1-rt-AP | GATTCACCATAACCACTGAGTCTTAG | qPCR |
| c23-1-rt-P | TCCACTACCTGCTGAATCCAAC | qPCR |
| c23-4-rt-AP | CTTGCTAGGGGTATCCGGTT | qPCR |
| c23-4-rt-P | TGGAAGTCTTGCTATCGTTACCT | qPCR |
| c26-2-rt-AP | GCTAATATTGGAAGCAACAGCAC | qPCR |
| c26-2-rt-P | TGATAATCTTCGGAAACCACTG | qPCR |
| c26-4-rt-AP | TACTTTAGGTTGCGGGTAAAGGT | qPCR |
| c26-4-rt-P | TTGTTTTCCTCGTCGCCAT | qPCR |
| c27-2-rt-AP | CTGACTCAAATGGGAGCCAAT | qPCR |
| c27-2-rt-P | GTCGTTGGGTTGTCCACACTAC | qPCR |
| c28-6-rt-AP | CGTCCAGTCAGTGTATGAAAAGTG | qPCR |
| c28-6-rt-P | GGAAGATGGTGTGGAATGAAAG | qPCR |
| c29-6-rt-AP | TTGATTGGTCCATACACGATTG | qPCR |
| c29-6-rt-P | TGACATCTACGCAAGAACCACT | qPCR |
| c3-3-rt-AP | GCATGCATAATCTGTATCGGTTG | qPCR |
| c3-3-rt-P | AGCAGCTTGTGCATTTTGATG | qPCR |
| c5-3-rt-AP | CGCTTTGAAAGCTGTTACATCAT | qPCR |
| c5-3-rt-P | ACACACGTTCCGAATCCTTTC | qPCR |
| c6-2-rt-AP | CCTCTTAGAAATAGTGTACGTGTCG | qPCR |
| c6-2-rt-P | AGAAGAGAGGACGGAAGCTGTC | qPCR |
| c6-6-rt-AP | TAGGGGTAGCACTGGCTCTTTC | qPCR |
| c6-6-rt-P | GGAATCCAGATGAACCGTGC | qPCR |
| β-tubulin-P | GACGCATGTCCATGAAGGAG | qPCR |
| β-tubulin-AP | CCAATGCAAGAAAGCCTTGC | qPCR |
| β-actin-P | TGGCACCACACCTTCTAC | qPCR |
| β-actlin-AP | CATGATCTGGGTCATCTTCT | qPCR |
| c26-4-dsRNA-P | TAATACGACTCACTATAGGTGGGAACATCATTTGGTGCA | dsRNA synthesis |
| c26-4-dsRNA-AP | TAATACGACTCACTATAGGTTAACTAGATTGTAGATAAGGAT | dsRNA synthesis |
| dsGFP-F | TAATACGACTCACTATAGGAAGGGCGAGGAGCTGTTCCCG | dsRNA synthesis |
| dsGFP-R | TAATACGACTCACTATAGGCAGCAGGACCATGTGATCGCGC | dsRNA synthesis |
| c26-4-HTb-P | ACGAGCTCACTAGTCGCGGCCGCTATGATGTTCTTCAGCAAATTACC | vector concentration |
| c26-4-HTb-AP | CTTGGTACCGCATGCCTCGAGTTAACTAGATTGTAGATAAGGATA | vector concentration |
| c2-1-HTb-P | ACGAGCTCACTAGTCGCGGCCGCTATGACGAGTACTCTACTGATGTT | vector concentration |
| c2-1-HTb-AP | CTTGGTACCGCATGCCTCGAGTCAATACCAATTTTGACGTGAG | vector concentration |
| c3-5-HTb-P | ACGAGCTCACTAGTCGCGGCCGCTATGGGAATGATCATTACAAA | vector concentration |
| c3-5-HTb-AP | CTTGGTACCGCATGCCTCGAGTTAACGTAAAAACTCCGTTA | vector concentration |
| c26-4-pUASTattB-P | TTCGTTAACAGATCTGCGGCCGCATGATGTTCTTCAGCAAATTACC | vector concentration |
| c26-4-pUASTattB-AP | TCCTCTAGAGGTACCCTCGAGTTAACTAGATTGTAGATAAGGATA | vector concentration |

Underline, T7 promoter sequences

**Table S8. Read statistics resulting from Illumina transcriptomic deep sequencing of parasitized *P. xylostella* larvae hemocytes.**

| Time pp | Replicate | Raw reads | Clean reads | CvBV-related Reads (ratio %) |
| --- | --- | --- | --- | --- |
| 6 h | 1 | 58,151,626 | 58,068,234 | 2,568,012 (4.42%) |
|  | 2 | 57,757,936 | 57,663,794 | 2,636,222 (4.57%) |
|  | 3 | 55,067,356 | 54,966,534 | 2,545,575 (4.63%) |
| 12 h | 1 | 59,815,090 | 59,677,350 | 1,139,220 (1.91%) |
|  | 2 | 57,973,350 | 57,846,924 | 1,769,146 (3.06%) |
|  | 3 | 60,988,612 | 60,869,968 | 1,709,806 (2.81%) |
| 24 h | 1 | 67,286,962 | 67,146,568 | 1,416,586 (2.11%) |
|  | 2 | 61,387,936 | 61,252,162 | 1,438,292 (2.35%) |
|  | 3 | 60,329,216 | 60,201,872 | 1,587,835 (2.64%) |
| Total reads |  | 538,758,084 | 537,693,406 | 16,810,694 (3.13%) |

**Table S9 FPKMs of CvBV genes expressed in *P. xylostella* hemocytes at 6 h pp.**

| **CvBV-ID** | **FPKMs** |
| --- | --- |
| CvBV-26-4 | 17842.6 |
| CvBV-3-5 | 16854.4 |
| CvBV-29-3 | 9159.72 |
| CvBV-7-6 | 8034.73 |
| CvBV-2-1 | 6966.06 |
| CvBV-23-5 | 6147.63 |
| CvBV-7-2 | 5855.37 |
| CvBV-7-1 | 5654.66 |
| CvBV-29-1 | 5554.12 |
| CvBV-23-4 | 4863.93 |
| CvBV-12-8 | 4689.64 |
| CvBV-29-7 | 4421.43 |
| CvBV-34-1 | 3868.98 |
| CvBV-11-1 | 3527.56 |
| CvBV-5-2 | 3370.79 |
| CvBV-26-3 | 3275.54 |
| CvBV-7-5 | 3184.57 |
| CvBV-3-3 | 2440.97 |
| CvBV-22-3 | 2435.23 |
| CvBV-5-1 | 2389.33 |
| CvBV-23-1 | 2348.28 |
| CvBV-5-3 | 2327.29 |
| CvBV-11-2 | 2100.34 |
| CvBV-34-3 | 2031.83 |
| CvBV-21-2 | 2025.11 |
| CvBV-3-2 | 1835.62 |
| CvBV-1-1 | 1606.08 |
| CvBV-23-3 | 1602.6 |
| CvBV-15-2 | 1574.9 |
| CvBV-18-2 | 971.985 |
| CvBV-32-1 | 944.436 |
| CvBV-16-4 | 631.382 |
| CvBV-22-2 | 569.661 |
| CvBV-4-5 | 530.386 |
| CvBV-11-7 | 520.8 |
| CvBV-34-2 | 505.903 |
| CvBV-34-5 | 473.782 |
| CvBV-29-4 | 446.964 |
| CvBV-15-1 | 419.962 |
| CvBV-26-1 | 403.133 |
| CvBV-24-8 | 350.495 |
| CvBV-8-1 | 301.794 |
| CvBV-11-6 | 250.012 |
| CvBV-3-1 | 239.82 |
| CvBV-16-1 | 239.165 |
| CvBV-21-3 | 233.575 |
| CvBV-23-2 | 233.38 |
| CvBV-6-4 | 230.737 |
| CvBV-12-5 | 226.887 |
| CvBV-29-5 | 200.604 |
| CvBV-11-4 | 191.136 |
| CvBV-27-2 | 185.372 |
| CvBV-18-1 | 184.376 |
| CvBV-4-2 | 176.479 |
| CvBV-26-2 | 172.448 |
| CvBV-18-4 | 157.059 |
| CvBV-12-6 | 150.853 |
| CvBV-16-2 | 130.724 |
| CvBV-6-1 | 126.487 |
| CvBV-10-2 | 112.84 |
| CvBV-27-1 | 111.941 |
| CvBV-30-1 | 107.658 |
| CvBV-14-6 | 105.427 |
| CvBV-12-7 | 99.4298 |
| CvBV-1-2 | 68.0031 |
| CvBV-3-6 | 64.7541 |
| CvBV-24-7 | 64.481 |
| CvBV-28-2 | 62.1497 |
| CvBV-10-1 | 61.919 |
| CvBV-18-3 | 57.9272 |
| CvBV-13-1 | 56.5336 |
| CvBV-4-3 | 56.2668 |
| CvBV-16-3 | 51.3893 |
| CvBV-6-6 | 49.3548 |
| CvBV-7-3 | 47.6755 |
| CvBV-34-4 | 40.594 |
| CvBV-7-4 | 40.0895 |
| CvBV-22-1 | 38.9722 |
| CvBV-11-3 | 38.6534 |
| CvBV-29-2 | 36.8063 |
| CvBV-33-1 | 36.056 |
| CvBV-14-1 | 32.8294 |
| CvBV-21-4 | 27.0099 |
| CvBV-14-4 | 26.3561 |
| CvBV-14-3 | 25.7287 |
| CvBV-4-1 | 23.9155 |
| CvBV-29-6 | 23.891 |
| CvBV-9-1 | 22.9583 |
| CvBV-14-5 | 20.2083 |
| CvBV-28-3 | 19.5831 |
| CvBV-4-7 | 18.0922 |
| CvBV-24-2 | 17.2146 |
| CvBV-14-7 | 17.1111 |
| CvBV-12-11 | 16.304 |
| CvBV-14-2 | 16.2833 |
| CvBV-12-4 | 16.1745 |
| CvBV-12-9 | 15.1036 |
| CvBV-28-1 | 12.2835 |
| CvBV-12-10 | 11.8944 |
| CvBV-17-1 | 11.2199 |
| CvBV-20-4 | 10.8848 |
| CvBV-20-1 | 10.6372 |
| CvBV-17-2 | 8.80073 |
| CvBV-12-3 | 7.82057 |
| CvBV-13-4 | 7.71184 |
| CvBV-3-4 | 7.47394 |
| CvBV-28-4 | 6.46075 |
| CvBV-21-1 | 5.47778 |
| CvBV-11-5 | 5.33497 |
| CvBV-9-2 | 4.90546 |
| CvBV-6-5 | 4.47408 |
| CvBV-24-10 | 3.85735 |
| CvBV-20-8 | 3.18469 |
| CvBV-13-2 | 3.15209 |
| CvBV-6-7 | 2.94808 |
| CvBV-9-3 | 2.93669 |
| CvBV-4-4 | 2.69156 |
| CvBV-2-2 | 2.66316 |
| CvBV-24-5 | 2.56646 |
| CvBV-12-2 | 2.52388 |
| CvBV-25-2 | 2.41107 |
| CvBV-20-2 | 1.98135 |
| CvBV-28-5 | 1.88603 |
| CvBV-35-1 | 1.61939 |
| CvBV-24-4 | 1.47314 |
| CvBV-13-3 | 1.41876 |
| CvBV-20-3 | 1.33867 |
| CvBV-28-6 | 1.24802 |
| CvBV-4-6 | 0.94715 |

**Table S10 FPKMs of CvBV genes expressed in *P. xylostella* hemocytes at 12 h pp**

| **CvBV-ID** | **FPKMs** |
| --- | --- |
| CvBV-2-1 | 18020.7 |
| CvBV-3-5 | 10644.4 |
| CvBV-12-8 | 8637.5 |
| CvBV-26-4 | 7839.62 |
| CvBV-23-5 | 6808.8 |
| CvBV-23-4 | 4486.55 |
| CvBV-21-2 | 3313.88 |
| CvBV-29-1 | 3091.52 |
| CvBV-5-3 | 2685.07 |
| CvBV-7-1 | 2510.62 |
| CvBV-5-1 | 2408.92 |
| CvBV-5-2 | 2257.33 |
| CvBV-29-7 | 2171.69 |
| CvBV-7-2 | 1876.58 |
| CvBV-23-1 | 1533.73 |
| CvBV-29-3 | 1478.68 |
| CvBV-11-2 | 1033.75 |
| CvBV-3-2 | 1000.98 |
| CvBV-3-3 | 964.194 |
| CvBV-26-3 | 859.437 |
| CvBV-34-1 | 842.984 |
| CvBV-7-6 | 747.933 |
| CvBV-34-3 | 648.38 |
| CvBV-22-3 | 633.111 |
| CvBV-7-5 | 572.15 |
| CvBV-18-2 | 548.358 |
| CvBV-23-3 | 531.974 |
| CvBV-11-1 | 444.856 |
| CvBV-1-1 | 429.205 |
| CvBV-15-2 | 389.873 |
| CvBV-27-2 | 314.218 |
| CvBV-4-5 | 301.572 |
| CvBV-30-1 | 284.511 |
| CvBV-23-2 | 203.957 |
| CvBV-34-5 | 201.388 |
| CvBV-4-2 | 198.87 |
| CvBV-12-6 | 181.725 |
| CvBV-15-1 | 171.704 |
| CvBV-24-8 | 169.664 |
| CvBV-32-1 | 163.28 |
| CvBV-11-6 | 160.833 |
| CvBV-34-2 | 153.911 |
| CvBV-16-4 | 147.93 |
| CvBV-12-7 | 136.027 |
| CvBV-14-6 | 130.14 |
| CvBV-18-1 | 126.921 |
| CvBV-21-3 | 121.617 |
| CvBV-12-5 | 107.603 |
| CvBV-3-1 | 100.526 |
| CvBV-26-1 | 97.6243 |
| CvBV-6-4 | 96.7697 |
| CvBV-16-1 | 92.3987 |
| CvBV-8-1 | 89.2654 |
| CvBV-22-2 | 87.9194 |
| CvBV-29-4 | 79.0536 |
| CvBV-18-4 | 69.2876 |
| CvBV-13-1 | 66.9844 |
| CvBV-27-1 | 64.8839 |
| CvBV-29-5 | 62.3623 |
| CvBV-4-3 | 56.623 |
| CvBV-3-6 | 55.3163 |
| CvBV-10-2 | 48.1924 |
| CvBV-9-1 | 41.1592 |
| CvBV-11-7 | 40.7468 |
| CvBV-6-6 | 39.8448 |
| CvBV-6-1 | 39.0833 |
| CvBV-18-3 | 37.8166 |
| CvBV-4-1 | 27.7819 |
| CvBV-4-7 | 27.1689 |
| CvBV-14-1 | 24.0896 |
| CvBV-14-3 | 21.6217 |
| CvBV-2-2 | 21.5701 |
| CvBV-12-4 | 20.7935 |
| CvBV-22-1 | 19.9411 |
| CvBV-14-4 | 19.0253 |
| CvBV-26-2 | 18.3026 |
| CvBV-10-1 | 17.3719 |
| CvBV-16-2 | 16.7145 |
| CvBV-17-1 | 15.1274 |
| CvBV-33-1 | 14.4986 |
| CvBV-11-4 | 13.9099 |
| CvBV-29-2 | 13.8117 |
| CvBV-11-3 | 13.6319 |
| CvBV-20-4 | 12.7494 |
| CvBV-12-9 | 12.2861 |
| CvBV-34-4 | 12.1807 |
| CvBV-17-2 | 11.9851 |
| CvBV-12-10 | 11.5598 |
| CvBV-12-11 | 11.2782 |
| CvBV-14-5 | 11.1395 |
| CvBV-12-3 | 9.99361 |
| CvBV-24-4 | 9.3254 |
| CvBV-9-2 | 9.28576 |
| CvBV-3-4 | 9.277 |
| CvBV-14-7 | 8.55929 |
| CvBV-7-3 | 7.65779 |
| CvBV-21-1 | 7.25631 |
| CvBV-29-6 | 7.01211 |
| CvBV-28-2 | 6.89066 |
| CvBV-14-2 | 6.50595 |
| CvBV-16-3 | 5.95774 |
| CvBV-1-2 | 5.18275 |
| CvBV-20-6 | 5.12608 |
| CvBV-4-4 | 4.841 |
| CvBV-20-2 | 4.79783 |
| CvBV-28-1 | 4.54225 |
| CvBV-24-10 | 4.47709 |
| CvBV-11-5 | 4.14544 |
| CvBV-20-1 | 3.54464 |
| CvBV-9-3 | 3.53065 |
| CvBV-24-3 | 3.44702 |
| CvBV-13-4 | 2.67516 |
| CvBV-7-4 | 2.38718 |
| CvBV-12-2 | 2.1583 |
| CvBV-28-4 | 2.0475 |
| CvBV-20-8 | 1.86248 |
| CvBV-28-3 | 1.81595 |
| CvBV-4-6 | 1.48023 |
| CvBV-6-3 | 1.45708 |
| CvBV-6-5 | 1.44918 |
| CvBV-6-7 | 1.44575 |
| CvBV-6-2 | 1.2313 |
| CvBV-25-2 | 0.917373 |
| CvBV-13-2 | 0.861879 |

**Table S11 FPKMs of CvBV genes expressed in *P. xylostella* hemocytes at 24 h pp**

| **CvBV-ID** | **FPKMs** |
| --- | --- |
| CvBV-2-1 | 31961.9 |
| CvBV-3-5 | 11822.9 |
| CvBV-26-4 | 6820.02 |
| CvBV-12-8 | 5756.2 |
| CvBV-23-5 | 4578.29 |
| CvBV-23-4 | 4553.95 |
| CvBV-7-1 | 3757.9 |
| CvBV-21-2 | 2132.23 |
| CvBV-29-1 | 1805.53 |
| CvBV-29-7 | 1741.83 |
| CvBV-5-1 | 1472.55 |
| CvBV-18-2 | 1468.92 |
| CvBV-7-2 | 1349.53 |
| CvBV-5-3 | 1247.42 |
| CvBV-3-2 | 1174.92 |
| CvBV-27-2 | 1113.45 |
| CvBV-3-3 | 987.812 |
| CvBV-23-1 | 956.678 |
| CvBV-11-2 | 809.345 |
| CvBV-23-3 | 746.514 |
| CvBV-5-2 | 739.415 |
| CvBV-26-3 | 582.67 |
| CvBV-7-6 | 493.979 |
| CvBV-29-3 | 430.93 |
| CvBV-30-1 | 365.672 |
| CvBV-23-2 | 275.922 |
| CvBV-3-1 | 256.015 |
| CvBV-15-2 | 254.814 |
| CvBV-18-1 | 251.584 |
| CvBV-24-8 | 250.289 |
| CvBV-4-5 | 245.204 |
| CvBV-22-3 | 228.884 |
| CvBV-7-5 | 201.095 |
| CvBV-34-1 | 172.666 |
| CvBV-6-4 | 167.428 |
| CvBV-11-1 | 162.353 |
| CvBV-1-1 | 150.624 |
| CvBV-4-2 | 146.221 |
| CvBV-11-6 | 130.095 |
| CvBV-14-6 | 125.267 |
| CvBV-34-3 | 119.714 |
| CvBV-12-6 | 118.215 |
| CvBV-8-1 | 117.429 |
| CvBV-6-6 | 106.22 |
| CvBV-32-1 | 102.304 |
| CvBV-18-3 | 98.9126 |
| CvBV-12-1 | 98.611 |
| CvBV-27-1 | 94.1773 |
| CvBV-12-7 | 93.2466 |
| CvBV-21-3 | 92.1736 |
| CvBV-15-1 | 84.8566 |
| CvBV-13-1 | 71.7861 |
| CvBV-9-1 | 71.5696 |
| CvBV-26-1 | 69.8872 |
| CvBV-3-6 | 67.5706 |
| CvBV-18-4 | 62.2556 |
| CvBV-12-5 | 62.1276 |
| CvBV-2-2 | 56.1045 |
| CvBV-10-2 | 55.2308 |
| CvBV-34-5 | 54.0173 |
| CvBV-34-2 | 49.6191 |
| CvBV-4-3 | 46.8877 |
| CvBV-6-1 | 45.4752 |
| CvBV-29-4 | 36.5281 |
| CvBV-11-7 | 36.1849 |
| CvBV-16-1 | 35.5404 |
| CvBV-29-5 | 31.526 |
| CvBV-24-2 | 30.9013 |
| CvBV-4-1 | 27.0965 |
| CvBV-22-2 | 24.4207 |
| CvBV-4-7 | 24.261 |
| CvBV-24-7 | 21.5662 |
| CvBV-10-1 | 21.2564 |
| CvBV-17-1 | 21.2047 |
| CvBV-12-10 | 20.238 |
| CvBV-14-5 | 20.2344 |
| CvBV-14-1 | 20.1473 |
| CvBV-21-4 | 19.7649 |
| CvBV-12-4 | 18.6115 |
| CvBV-16-4 | 16.3371 |
| CvBV-14-3 | 15.7586 |
| CvBV-17-2 | 13.9679 |
| CvBV-9-2 | 13.7592 |
| CvBV-26-2 | 11.5688 |
| CvBV-13-7 | 11.1921 |
| CvBV-7-3 | 10.8817 |
| CvBV-14-4 | 10.4975 |
| CvBV-22-1 | 10.4705 |
| CvBV-14-7 | 9.68238 |
| CvBV-20-5 | 9.59624 |
| CvBV-11-5 | 9.39998 |
| CvBV-11-4 | 9.16438 |
| CvBV-12-11 | 8.75118 |
| CvBV-12-9 | 8.61388 |
| CvBV-12-3 | 8.34456 |
| CvBV-3-4 | 8.30177 |
| CvBV-28-1 | 8.05426 |
| CvBV-20-4 | 7.94337 |
| CvBV-14-2 | 7.42938 |
| CvBV-16-2 | 7.17297 |
| CvBV-9-3 | 6.65012 |
| CvBV-29-2 | 6.33102 |
| CvBV-28-3 | 5.93307 |
| CvBV-21-1 | 5.31781 |
| CvBV-24-4 | 5.08524 |
| CvBV-7-4 | 4.79605 |
| CvBV-20-8 | 4.47892 |
| CvBV-28-2 | 4.21259 |
| CvBV-16-3 | 4.00663 |
| CvBV-11-3 | 3.63616 |
| CvBV-12-2 | 3.62957 |
| CvBV-33-1 | 3.42336 |
| CvBV-6-5 | 3.20853 |
| CvBV-24-10 | 3.18481 |
| CvBV-29-6 | 3.15694 |
| CvBV-24-3 | 3.07884 |
| CvBV-4-4 | 3.07195 |
| CvBV-1-2 | 3.03622 |
| CvBV-20-1 | 2.93532 |
| CvBV-6-7 | 2.86379 |
| CvBV-34-4 | 2.74099 |
| CvBV-20-6 | 2.3753 |
| CvBV-28-4 | 2.26249 |
| CvBV-13-4 | 2.25947 |
| CvBV-4-6 | 2.24623 |
| CvBV-25-2 | 2.18752 |
| CvBV-20-2 | 2.18441 |
| CvBV-6-3 | 1.94659 |
| CvBV-24-5 | 1.49828 |
| CvBV-6-2 | 1.3153 |
| CvBV-25-1 | 1.15051 |
| CvBV-35-1 | 0.90141 |
| CvBV-13-2 | 0.876314 |


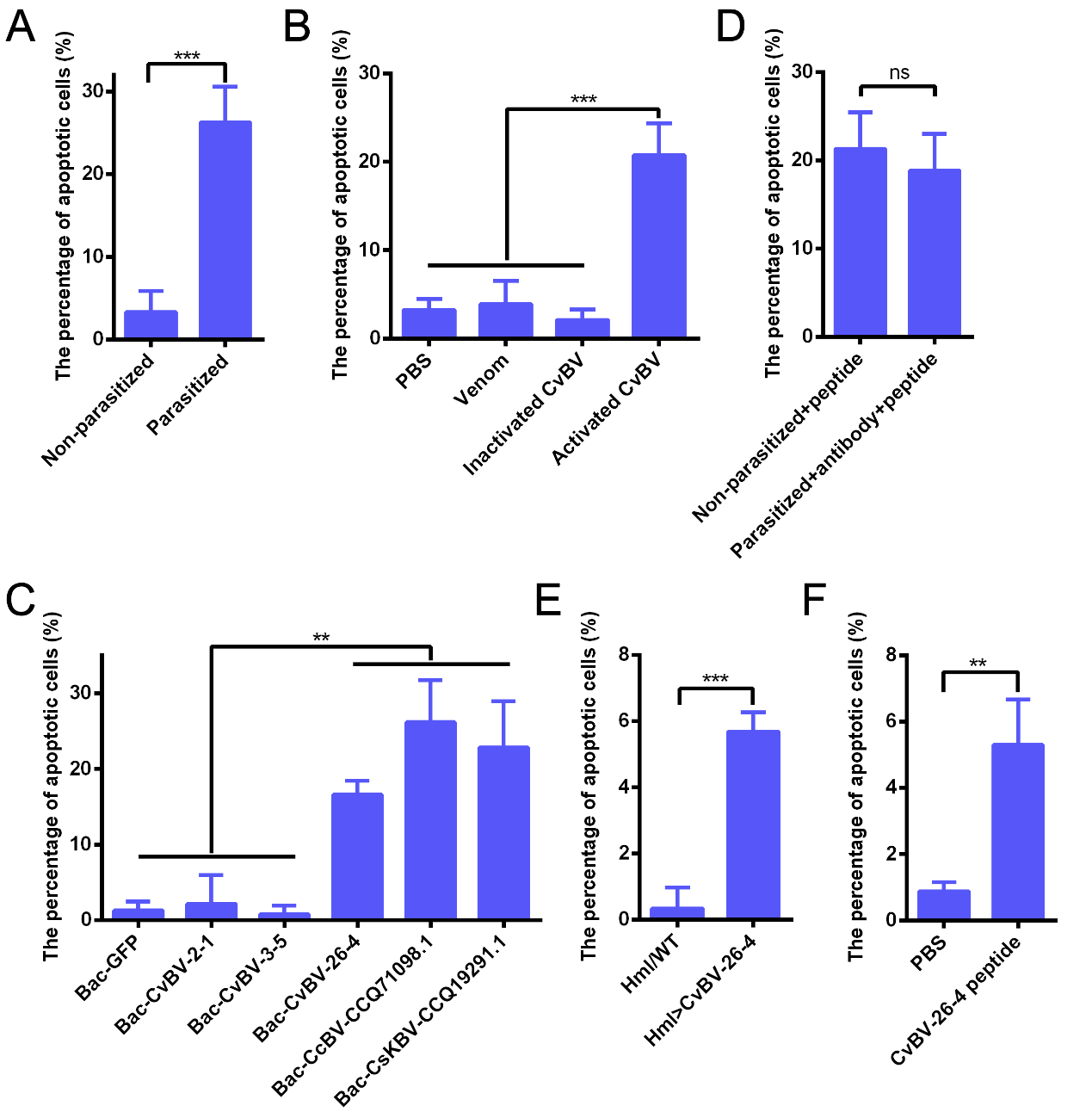


**Figure S1** The percentage of apoptotic hemocytes from *P. xylostella* and *D. melanogaster*. **A:** The percentage of apoptotic hemocytes from *P. xylostella* at 24 h pp. **B:** The percentage of apoptotic hemocytes from *P. xylostella* at 24 h post-injection of PBS, venom, inactivated and activated CvBV. **C:** The percentage of apoptotic hemocytes from *P. xylostella* at 24 h post infected by modified baculoviruses (Bac-GFP, Bac-CvBV-2-1, Bac-CvBV-3-,5, Bac-CvBV-26-4, Bac-CcBV-CCQ71098.1 and Bac-CsKBV-CCQ19291.1).**D:** The percentage of apoptotic hemocytes from *P. xylostella* at 24 h post-injection of the CvBV-26-4 peptide and from parasitized *P. xylostella* at 24 h post-injection of the mixture of CvBV-26-4 antibody and CvBV-26-4 peptide. **E:** The percentage of apoptotic hemocytes from *Drosophila larvae* that ectopically expressed *CvBV-26-4*. **F:** The percentage of apoptotic hemocytes from 3^rd^ instar *Drosophila* larvae (*W^1118^*) at 24 h post-injection of 0.1 μg CvBV BAP peptide (injection of PBS was used as a negative control). Differences among samples were tested with Tukey-test (n=5, **: statistical difference for p < 0.01, ***: statistical difference for p < 0.001, ns: no signiﬁcance).





**Figure S2** Parasitization and injection of BAP peptide induce the caspase activity of *P. xylostella* hemocytes. **A**: The caspase 3/7 activity of *P. xylostella* hemocytes at 24 h pp. **B**: The caspase 3/7 activity of *P. xylostella* hemocytes at 24 h post injection of 0.1 μg CvBV BAP peptide. For the enzyme assay the hemocytes from one larva were used. The data represent the mean of 15 replicates in duplicates ± SE. Differences among samples were tested with Tukey-test (**: statistical difference for p < 0.01, ***: statistical difference for p < 0.001).


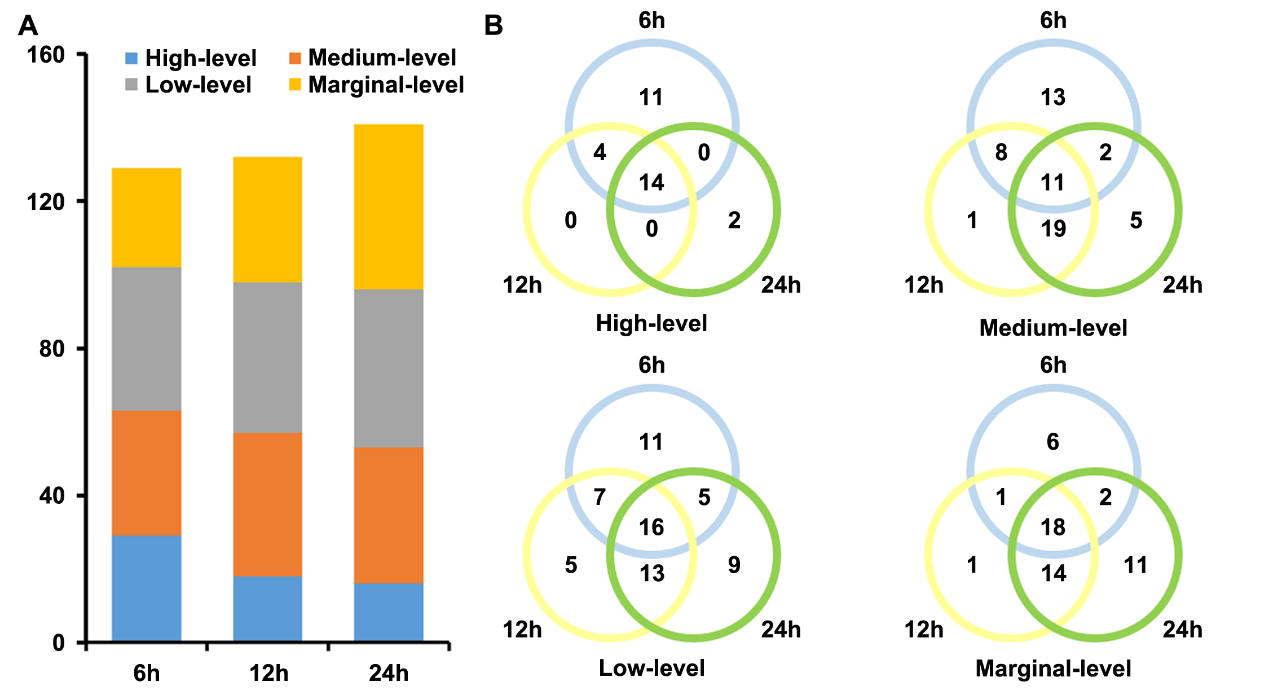


**Figure S3 Characteristic of CvBV genes transcribed in parasitized *P. xylostella* hemocytes.** **A:** The numbers of CvBV genes transcribed at different levels in *P. xylostella* hemocytes at 6 h, 12 h, and 24 h pp by *C. vestalis*. **B:** The relationship of CvBV genes transcribed at the same level at 6 h, 12 h, and 24 h pp by *C. vestalis.*


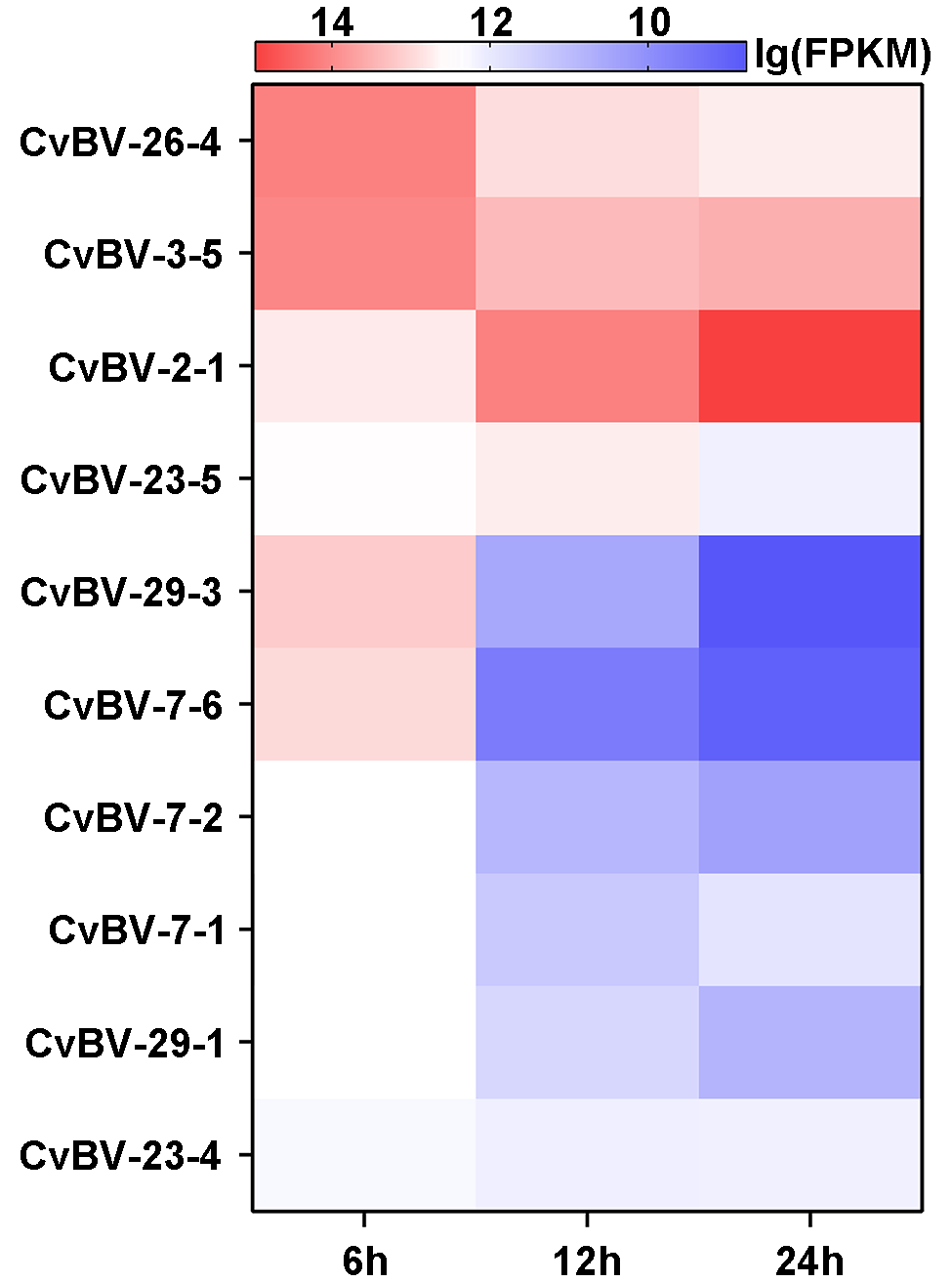


**Figure S4 Heat map illustrating the expression levels of top 10 CvBV genes at 6 h, 12 h, and 24 h pp.** The abscissa represents different times post parasitization and the ordinate represents different CvBV genes.


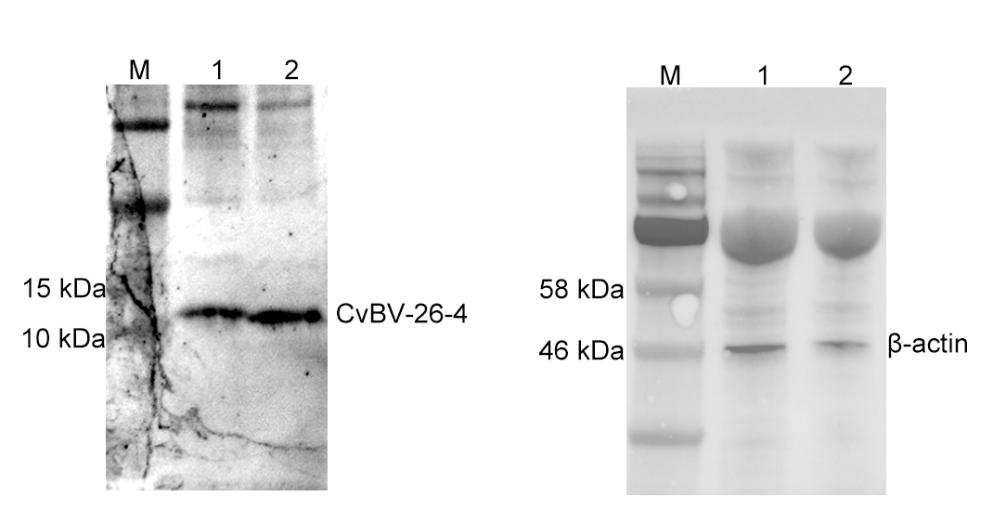


**Fig S5. Western blot of CvBV-26-4 in host hemolymph 24 h after the interference of *CvBV-26-4*.** A band in the size range of 13 kDa that corresponds to the predicted CvBV-26-4 protein. β-actin was used to show equal protein loading. M: marker, lane 1: *dsGFP*, and lane 2: *dsCvBV-26-4*.


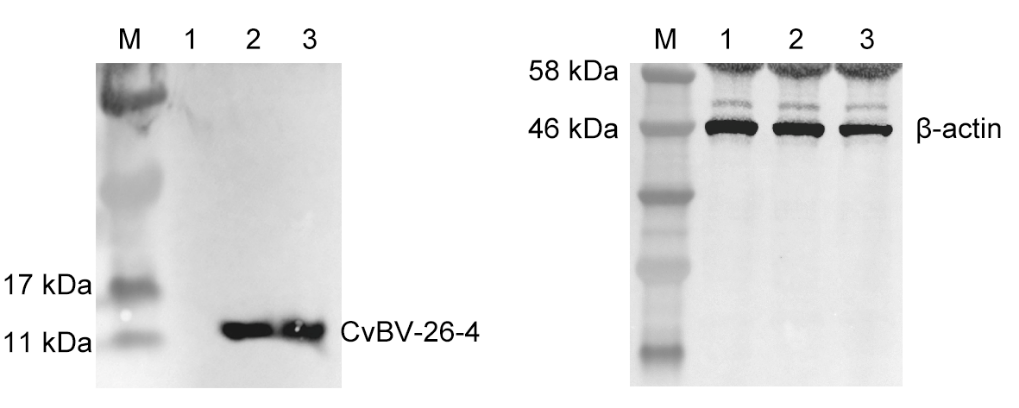


**Fig S6. Western blot of CvBV-26-4 protein in the hemolymph of *P. xylostella* at 24 h post infected by baculoviruses.** A band in the size range of 13 kDa that corresponds to the predicted CvBV-26-4 protein. β-actin was used to show equal protein loading. M: marker, lane 1: Bac-GFP (as negative control), lane 2: Bac-CvBV-26-4, and parasitized (as positive control).


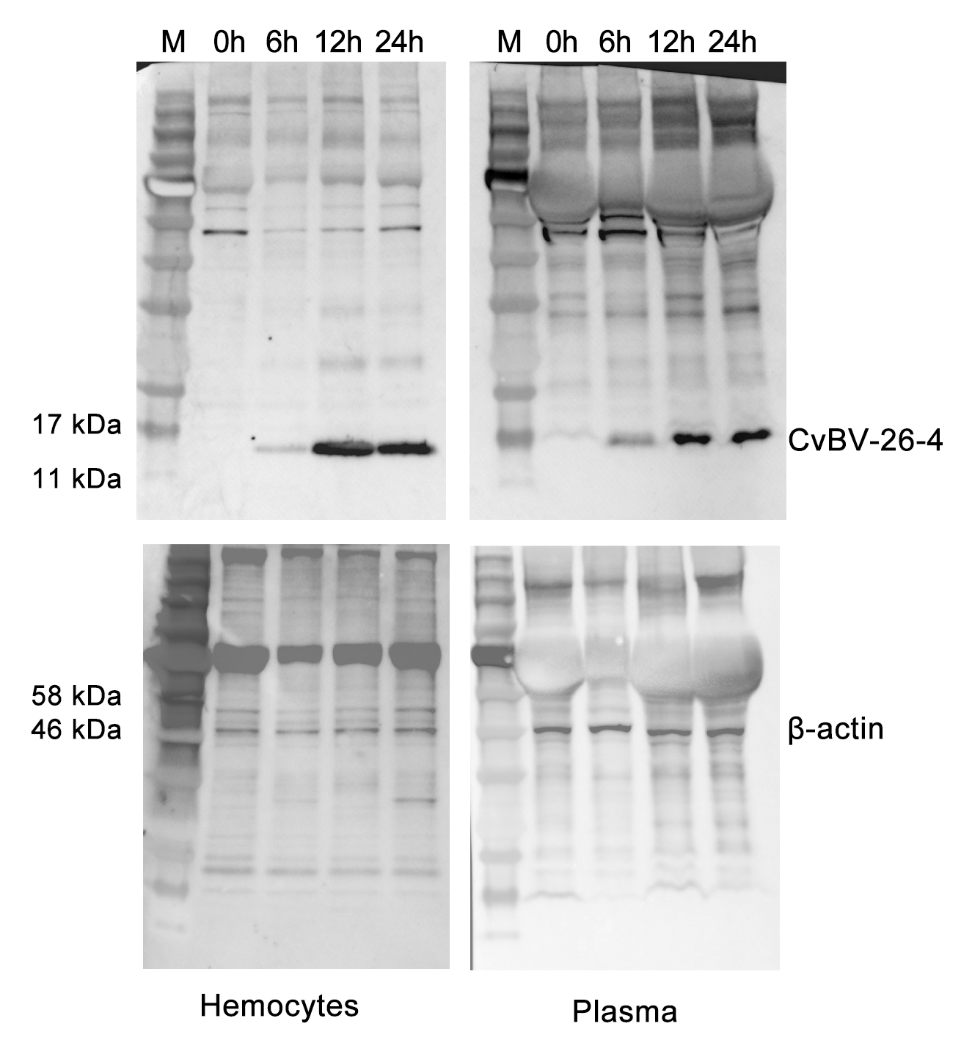


**Fig S7.** **Western blot of CvBV-26-4 protein in the hemocytes and plasma of *P. xylostella* at 6 h, 12 h, and 24 h pp.** A band in the size range of 13 kDa that corresponds to the predicted CvBV-26-4 protein. β-actin was used to show equal protein loading.


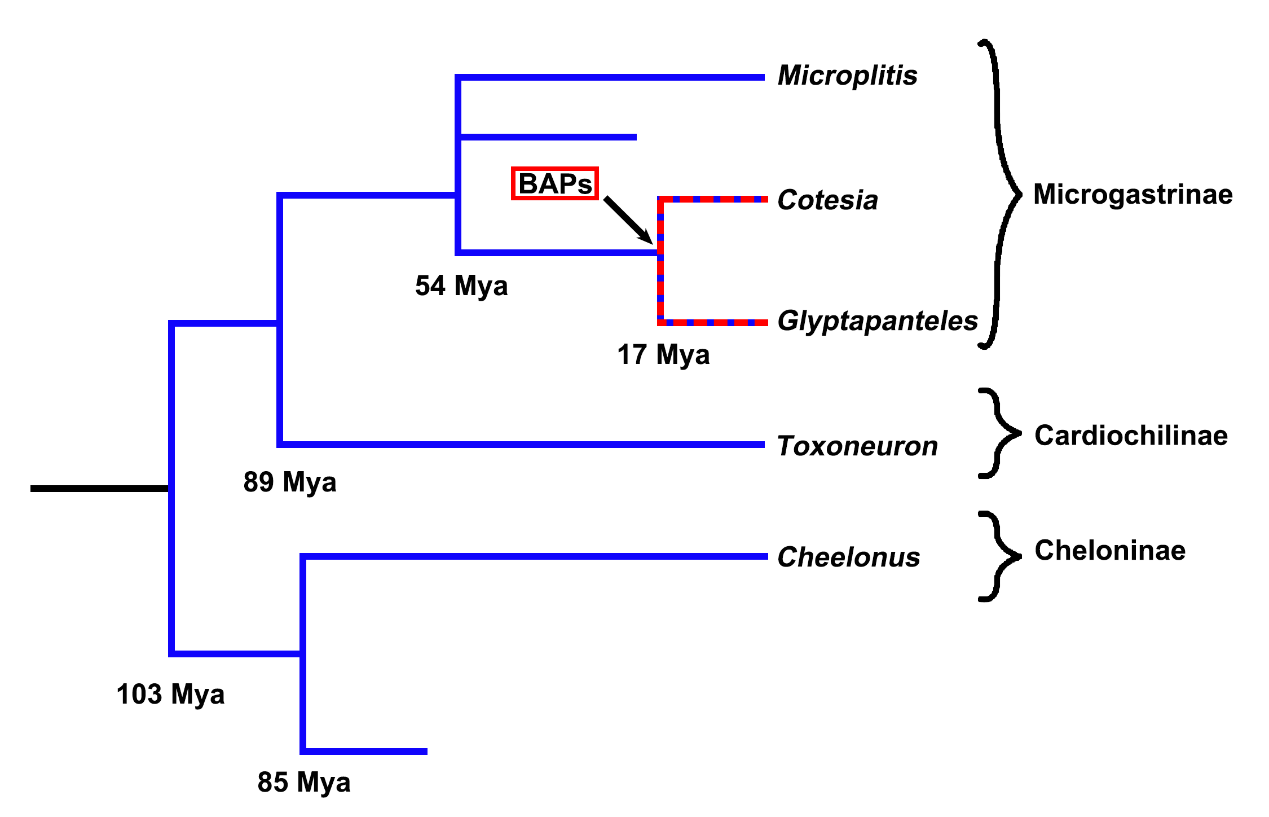


**Fig S8. A proposed evolutionary scenario for the acquisition of BAP in bracoviruses.** Key dates (in millions of years, Mya) for the origin of braconid microgastroid subfamilies are from Murphy et al. (2008). BAPs have only been identified in *Cotesia* bracoviruses and BV-carrying wasps of the genus *Glyptapanteles*. A proposed evolutionary scenario is that the acquisition of BAPs occurred between 54 Mya and 17 Mya.
